# Electrifying Hydroformylation Catalysts Exposes Voltage-Driven C–C Bond Formation

**DOI:** 10.1021/jacs.4c02992

**Published:** 2024-06-10

**Authors:** Joy S. Zeng, Emma L. Cosner, Spencer P. Delgado-Kukuczka, Chenyu Jiang, Jason S. Adams, Yuriy Román-Leshkov, Karthish Manthiram

**Affiliations:** † Department of Chemical Engineering, Massachusetts Institute of Technology, 77 Massachusetts Avenue, Cambridge, Massachusetts 02139, United States; ‡ Division of Chemistry and Chemical Engineering, California Institute of Technology, Pasadena, California 91125, United States

## Abstract

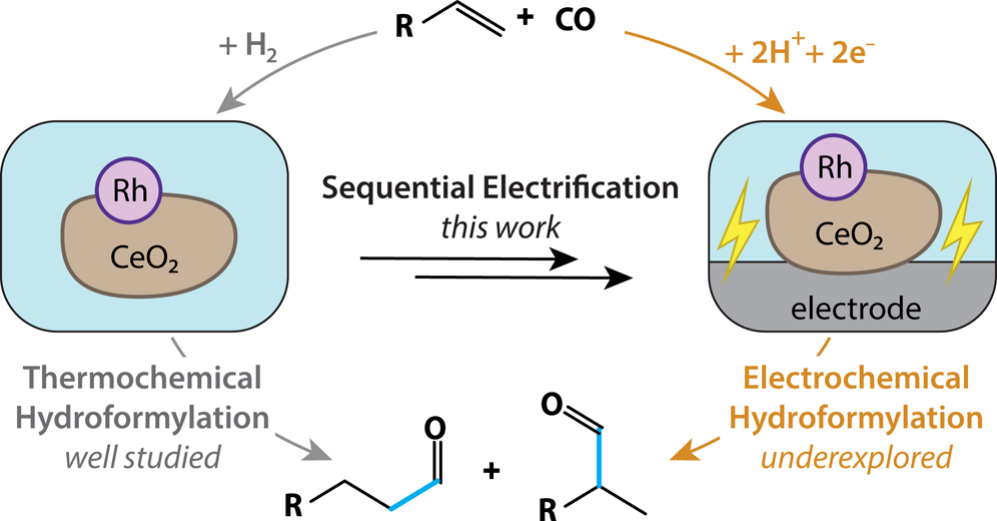

Electrochemical reactions can access a significant range of driving forces under operationally mild conditions and are thus envisioned to play a key role in decarbonizing chemical manufacturing. However, many reactions with well-established thermochemical precedents remain difficult to achieve electrochemically. For example, hydroformylation (thermo-HFN) is an industrially important reaction that couples olefins and carbon monoxide (CO) to make aldehydes. However, the electrochemical analogue of hydroformylation (electro-HFN), which uses protons and electrons instead of hydrogen gas, represents a complex C–C bond-forming reaction that is difficult to achieve at heterogeneous electrocatalysts. In this work, we import Rh-based thermo-HFN catalysts onto electrode surfaces to unlock electro-HFN reactivity. At mild conditions of room temperature and 5 bar CO, we achieve Faradaic efficiencies of up to 15% and turnover frequencies of up to 0.7 h^–1^. This electro-HFN rate is an order of magnitude greater than the corresponding thermo-HFN rate at the same catalyst, temperature, and pressure. Reaction kinetics and *operando* X-ray absorption spectroscopy provide evidence for an electro-HFN mechanism that involves distinct elementary steps relative to thermo-HFN. This work demonstrates a step-by-step experimental strategy for electrifying a well-studied thermochemical reaction to unveil a new electrocatalyst for a complex and underexplored electrochemical reaction.

## 1 Introduction

**Figure 1:**
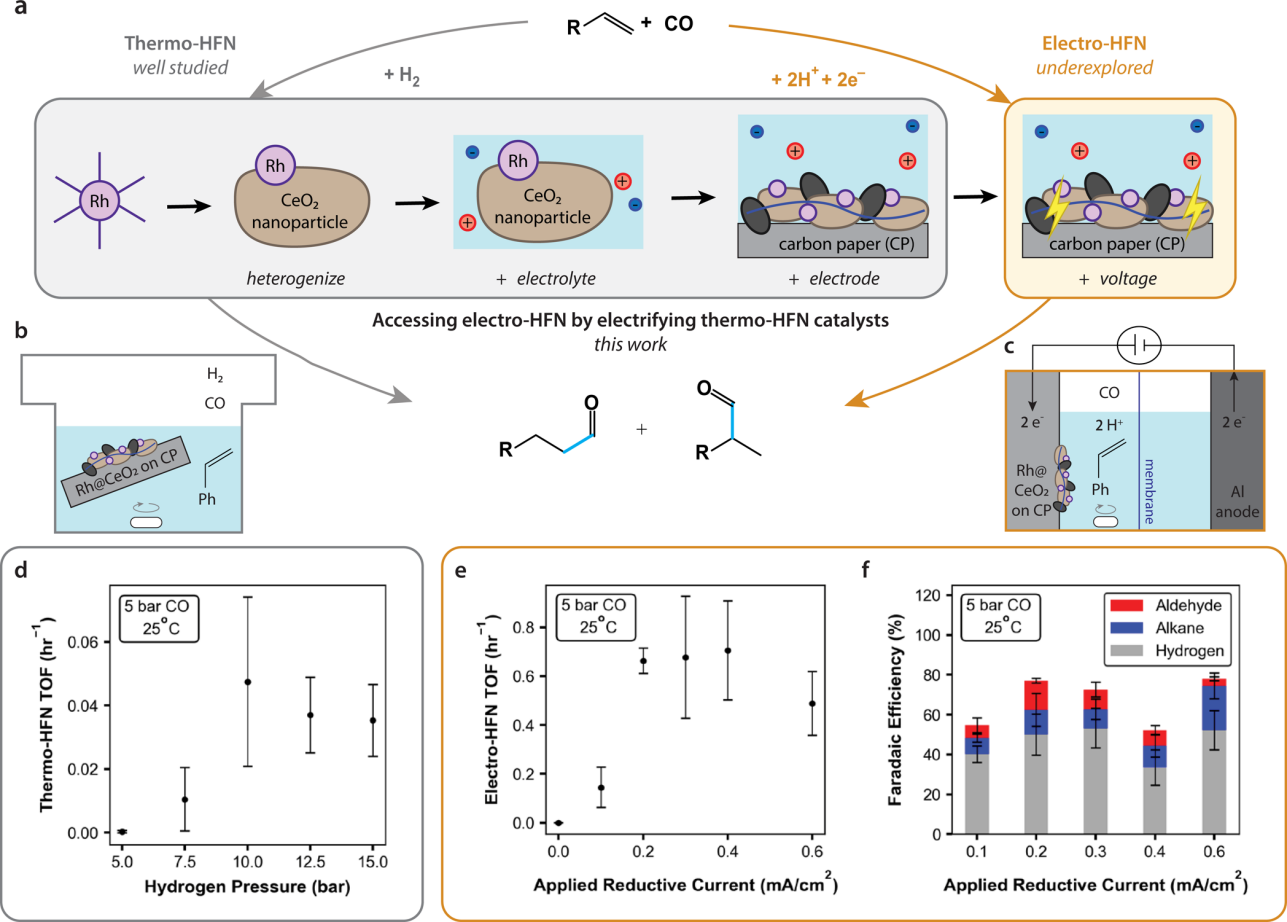
Electrifying a thermo-HFN catalyst to access electro-HFN reactivity. (a) Thermochemical and electrochemical hydroformylation (HFN) reactions. In this work, a thermo-HFN catalyst was sequentially electrified to achieve electro-HFN. Black ovals represent carbon black and blue lines represent Nafion binder. (b) Batch reaction setup within a pressurized Parr reactor used for thermo-HFN experiments. (c) Batch two-compartment, two-electrode electro-HFN cell configuration for elevated pressure electro-HFN experiments. (d) Thermo-HFN turnover frequency (TOF) as a function of partial H_2_ pressure. (e) Electro-HFN TOF as a function of applied reductive current. (f) Electro-HFN product distribution, given as Faradaic efficiency, as a function of applied reductive current. All data were collected at 25 °C, 5 bar CO, 0.52 M styrene, 0.1 M TBAOTf, 25 mM HOTf in a 50% v/v IPA/H_2_O mixture. Error bars represent standard deviation with *n* ≥ 3.

Electrochemical reactions leverage voltage as a potent driving force that can be renewably sourced. Although electrified manufacturing is envisioned to provide sustainable routes to important fuels and chemicals,^1−3^ many critical industrial reactions lack well-developed electrochemical alternatives. For example, the formation of carbon–carbon (C–C) bonds between olefins and carbon monoxide (CO) is an important reaction for which electrocatalytic routes are underexplored. The current thermochemical route is hydroformylation (thermo-HFN), which adds CO and H_2_ to olefins to generate aldehydes (Figure 1a). Because olefins are among the highest volume bulk chemicals^4^ and aldehydes are versatile intermediates for many products such as detergents and plastics,^4−7^ thermo-HFN is performed at a global scale of over 10 million tons/year.^4−6^

However, forming C–C bonds between olefins and CO is challenging in heterogeneous electrocatalysis. Related reactivity involving coupling between CO and ethylene is speculated to be a key step in forming three-carbon (C3) products such as propanol during CO_2_ and CO electroreduction.^8,9^ However, even with state-of-the-art Cu-based catalysts,^8,10^ the complex mixture of products formed has made it challenging to both understand reaction mechanisms and design catalysts that steer selectivity to one product.^8,11,12^ Additionally, the direct electrochemical analogue of hydroformylation (electro-HFN), in which protons and electrons replace the hydrogen gas used in thermo-HFN (Figure 1a), remains highly underdeveloped. To our knowledge, the first and only instance of electro-HFN was reported in 1997. The work featured spontaneous half reactions that required elevated temperatures and H_2_ as a reactant; additionally, externally applied voltage was not able to improve the observed performance.^13^ Thus, there is still a lack of catalysts that can facilitate voltage-driven C–C bond formation between olefins and CO.

We hypothesized that catalysts for electro-HFN could be designed via precedent from thermo-HFN catalysis. We show that electrifying a CeO_2_-supported Rh catalyst already known to be competent for thermo-HFN reveals a reaction system that is able to access two chemically similar reactions that use either voltage and protons (i.e., electro-HFN) or H_2_ gas (i.e., thermo-HFN). Within such a system, we show that accessing the voltage-driven electro-HFN reaction, beyond simply circumventing the need for H_2_ as a reactant, also mitigates the need for elevated temperatures (90–120 °C) that are typically used in thermo-HFN.

## 2 Results and Discussion

### 2.1 Sequentially Electrifying Thermo-HFN

We sequentially electrified thermo-HFN by implementing changes to typical organometallic thermo-HFN systems to make them more amenable for heterogeneous electrocatalysis. This involved synthesizing heterogenized catalytic sites, using ionically conductive reaction media, and depositing catalysts on electrode surfaces (Figure 1a).

For synthesizing heterogenized active sites, we followed the literature precedent showing that Rh atoms on metal oxide supports are active for hydroformylation.^14,15^ Based on initial screening of CeO_2_, TiO_2_, and Al_2_O_3_ as possible metal oxide supports, we found that Rh deposited on CeO_2_ (Rh@CeO_2_) was the most amenable to electrification (Supporting Information Section 2.2.1). To synthesize Rh@CeO_2_, we used wetness impregnation to deposit 1.8 wt % Rh (as determined by inductively coupled plasma mass spectrometry or ICP-MS) onto ∼20 nm sized cerium(IV) oxide nanoparticles (Figure S5). The final powder catalyst contained about 25% Ce^3+^ surface defects, as determined via X-ray photoelectron spectroscopy (XPS, Figure S6b). Additionally, the powder X-ray diffraction (pXRD) spectrum of Rh@CeO_2_ appeared similar to that of pure CeO_2_ (Figure S7), suggesting small and well-dispersed Rh species. The Rh@CeO_2_ powder catalyst was confirmed to be active for thermo-HFN of a model styrene substrate under typical thermo-HFN conditions:^14^ octane solvent, 80 °C, 5 bar CO, and 5 bar H_2_ (Figure S9a).

Then, while using the same catalyst, we moved to protic and ionically conductive solvent conditions that would eventually be required for electro-HFN. For the solvent, mixtures of water and alcohols allow thermo-HFN to proceed relatively unaffected, whereas other common solvents for organic electrochemistry, such as acetonitrile and *N*,*N*-dimethylformamide, interfere with reactivity (Figure S9a). For the salt and proton source, we saw favorable reactivity in the presence of triflate-containing salts and acids, particularly tetrabutylammonium triflate (TBAOTf) and triflic acid (HOTf) (Figure S9b).^16^

Finally, we deposited the Rh@CeO_2_ catalyst, along with carbon black and Nafion, onto carbon paper electrodes. Electro-HFN was performed in a two-compartment electrochemical cell with no H_2_ gas supplied (Figure 1c). We first performed electro-HFN at elevated temperatures (80 °C) and pressures (5 bar) to parallel typical thermo-HFN conditions. We found that some electro-HFN occurred at these elevated temperatures but at rates orders of magnitude below what we observed thermochemically (Figure S10). Surprisingly, electro-HFN rates and selectivities increased at ambient temperature (*vide infra*). Thus, our following discussions focus on experiments at ambient temperature.

One might expect that for the electrochemical reaction, conductivity via the ostensibly insulating CeO_2_ support could be limiting. However, under reducing conditions, ceria becomes more electronically conductive via electron hopping through lattice defects,^16^ and there are examples of immobilized nanoscale ceria with electrochemically accessible surface Ce^4+^/Ce^3+^ redox transitions.^17,18^ Furthermore, even in our as-synthesized catalyst, we observed the presence of Ce^3+^ defects in XPS. Nonetheless, we did incorporate carbon black into our electrodes to help mitigate possible conductivity issues. Finally, *operando* X-ray spectroscopy is consistent with a significant fraction of Rh sites being electrochemically accessible (*vide infra*), and more detailed discussion can be found in Supporting Information Section 2.11.

### 2.2 Demonstration of Electro-HFN

Electro-HFN of styrene at ambient temperature (25 °C) yielded the branched aldehyde product 2-phenylpropanal. During galvanostatic experiments at 5 bar CO, the turnover frequency (TOF), or rate per site (approximated with the total number of Rh atoms deposited), of electro-HFN reached a maximum of ∼0.7 h^–1^ at an applied current of −0.4 mA/cm^2^ (Figure 1e). When evaluating the same catalyst deposited on carbon paper within a thermochemical batch reactor that used H_2_ instead of applied voltage (Figure 1b), the maximum observed TOF was more than 10× lower (<0.05 h^–1^), even across a range of H_2_ pressures ranging from 5 to 15 bar (Figure 1d).

The maximum observed Faradaic efficiency (FE), or selectivity per electron, for electro-HFN was ca. 15%, which was observed at −0.2 mA/cm^2^, where other major side reactions included hydrogenation to yield ethylbenzene (13% FE) and the hydrogen evolution reaction (HER) (50% FE) (Figure 1f). Several other styrene-derived side products, including styrene dimerization products as well as unidentified organic species, were also observed. These side products likely account for the remaining 20–40% of FE (Supporting Information Section 2.3).

With respect to regioselectivity, we did not observe any linear aldehyde (3-phenylpropanal) for either thermo-HFN or electro-HFN at 25 °C. However, we did qualitatively observe more linear selectivity for both reactions at 80 °C, consistent with precedent that linear selectivity increases with increasing temperature.^19^ Additional regioselectivity discussion is included in Supporting Information Section 2.2.3. Finally, these data represent low-conversion experiments, with percent product yield typically less than 1%.

We then confirmed that electro-HFN activity occurred at heterogeneous sites because heterogeneous thermo-HFN catalysts can suffer from metal leaching.^20^ Although we did detect leached Rh in the electrolyte after electrolysis (up to 2% of deposited Rh after 2 h via ICP-MS, Figure S14a), an electrochemical “filtration test” suggests that the dissolved Rh species do not significantly contribute to catalysis: halfway through an electrolysis, swapping out the catalyst-loaded electrode with a blank carbon paper and reusing the same electrolyte completely stopped reaction progress (Figure S14b).

Next, we confirmed which reactions were associated with Rh-active sites by measuring the electro-HFN partial current, hydrogenation partial current, and total current as a function of Rh@CeO_2_ particle loading. We found that both electro-HFN and total current monotonically increased with increasing particle loading, whereas hydrogenation rates decreased with particle loading (Figure S15a). Electrodes with bare CeO_2_ nanoparticles or with only carbon black displayed a fair amount of background current and were active for hydrogenation but were not active for electro-HFN (Figure S15b). These results suggest that electro-HFN principally occurs on Rh-containing sites but that the same is not necessarily true for hydrogenation and other background reactions. Our observation that hydrogenation is mostly associated with a site different from that of electro-HFN surprisingly contrasts with what is understood for thermo-HFN, where hydrogenation is a side pathway within the hydroformylation mechanism.^5,6,21^

### 2.3 *Operando* Catalyst Characterization

We used X-ray absorption spectroscopy (XAS) at the Rh K-edge to characterize Rh valency (via X-ray absorption near-edge structure or XANES) and Rh coordination (via extended X-ray absorption fine structure or EXAFS) under both *ex situ* and *operando* conditions.^22^

**Figure 2:**
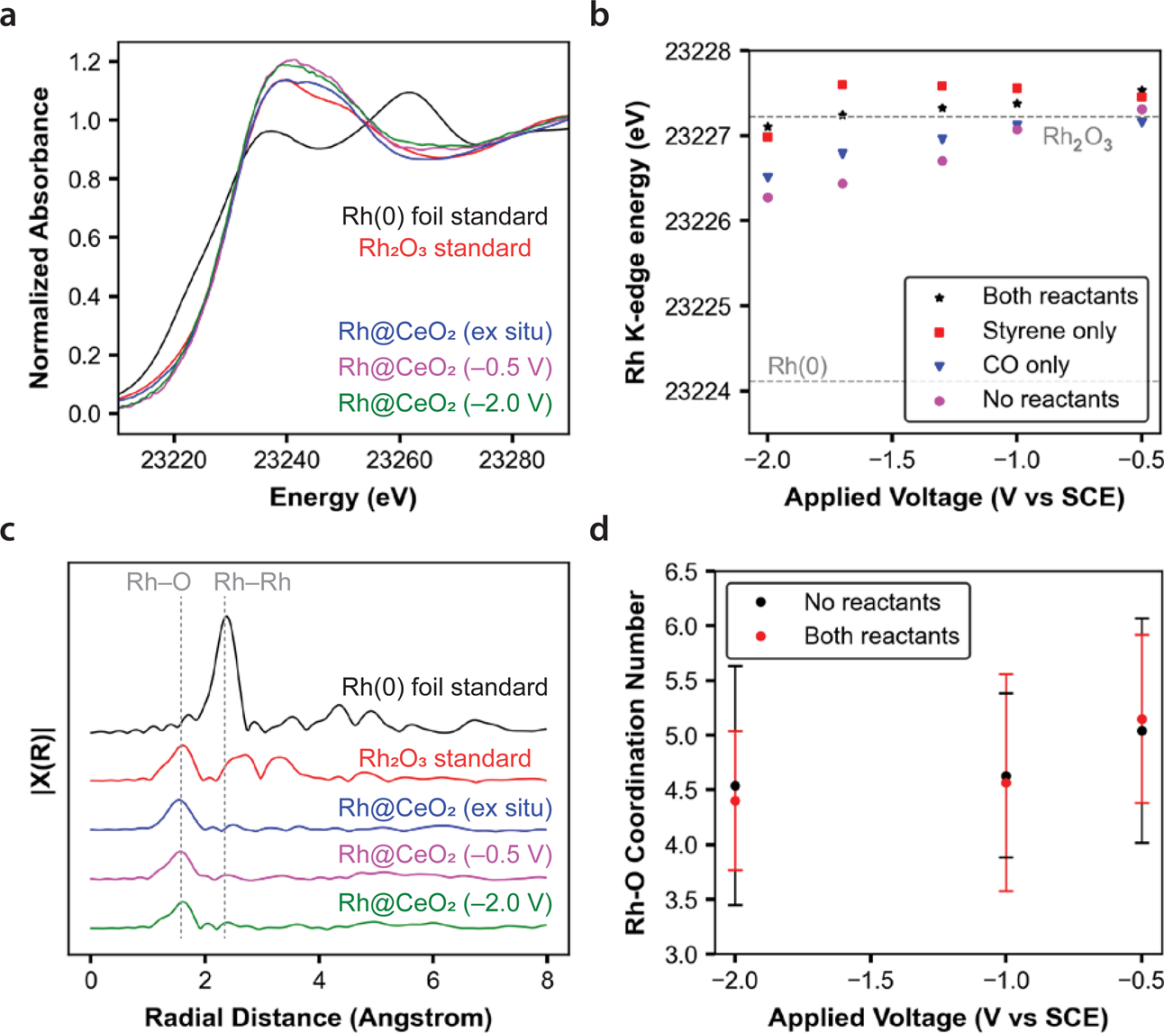
XAS. (a) Normalized XANES data zoomed in on rising edge, shown for *ex situ* and *operando* samples as well as known rhodium-containing standards. (b) Processed *operando* XANES data showing Rh K-edge energy as a function of applied potential (black stars), as well as control experiments without CO and/or styrene. (c) R-space EXAFS spectra of *ex situ* and *operando* samples, as well as known rhodium-containing standards. (d) Processed *operando* EXAFS data showing fitted Rh–O coordination numbers as a function of applied potential (red circles), as well as a control condition with neither CO nor styrene (black circles). Error bars in (d) represent fitting errors given by the Artemis fitting software. All data collected at ambient temperature and pressure in electrolyte composed of 0.1 M TBAOTf and 25 mM HOTf in IPA/H_2_O. Voltages reported vs SCE.

*Ex situ* measurements on the as-synthesized catalyst show the presence of hexacoordinate Rh(III). The XANES spectrum of the Rh@CeO_2_ catalyst is similar to that of a Rh_2_O_3_ standard, suggesting similar Rh oxidation states of +3 (Figure 2a). The Rh@CeO_2_ EXAFS spectrum shows a strong contribution from a first shell Rh–O scattering path with no visible Rh–Rh scattering contribution (Figure 2c). EXAFS fitting^23^ of the Rh–O scattering path shows that, within fitting error, both the Rh@CeO_2_ powder catalyst (CN_Rh–O_ = 5.8 ± 1.5) and the Rh_2_O_3_ standard (CN_Rh–O_ = 6) have the same Rh–O coordination number. These observations are consistent with either small Rh_2_O_3_ clusters or single Rh(III) atoms on the ceria surface and are consistent with the pXRD data that suggest small and well-dispersed Rh moieties on CeO_2_ (*vide supra*). Literature precedent indicates that at ∼2 wt % loadings, Rh deposited on similar metal oxide nanoparticles yields small clusters.^24^ In either case, the as-synthesized Rh(III) sites appear to be fully coordinated to either lattice or terminal oxygen atoms.

*Operando* measurements show that on average, the Rh@CeO_2_ catalyst remains as Rh(III) but coordinates fewer oxygen atoms under the reaction conditions. *Operando* XANES shows that under operating conditions with all reactants present, the Rh K-edge energy still reflects a Rh(III) oxidation state and does not change substantially in response to applied potential (Figure 2a and black stars in b). The Rh(III) assignment is in surprising contrast to the Rh(I) state that is expected from both homogeneous and heterogeneous thermo-HFN precedents.^5,21,25^*Operando* EXAFS shows that the Rh–O coordination number decreases to around 4.5–5 under reaction conditions, which suggests availability of open sites for catalysis (Figure 2c,d). We found no changes in fitted Rh–O bond lengths with applied potential (Figure S17b).

Notably, the XANES data indicates that the Rh sites are electrochemically accessible. When neither CO nor styrene are present, the Rh K-edge energy responds to applied potential (Figure 2b, purple dots), indicating that a significant fraction of the Rh is able to receive electron transfers from the electrode. This observed Rh reduction is not reversible (Figure S17a). The electrochemical accessibility of Rh sites should depend principally on the physical composition of the electrode and not on whether CO and styrene are present. Thus, the observation that Rh sites are electrochemically accessible under any condition suggests that even during *operando* conditions where no voltage-dependent changes in Rh valency are observed (Figure 2b, black stars), the Rh sites are also electrochemically accessible. We hypothesize that the presence of CO and styrene might attenuate the response of Rh valency to applied potential by making the Rh center itself harder to reduce, preventing reductive sintering,^26^ or acting as acceptors for the electrons that would otherwise go toward reducing Rh^27,28^ (Supporting Information Section 2.11.2).

In summary, the *operando* XAS data indicate that the resting state of the Rh catalyst is in a +3 oxidation state, coordinated to 4–5 oxygen ligands, electrochemically accessible, and weakly perturbed by changes in applied potential.

### 2.4 Evidence against H_2_ as a Mechanistic Intermediate

To investigate the mechanism of electro-HFN, we first considered the possibility that electro-HFN occurs via an indirect mechanism with H_2_ as an intermediate. In this case, the role of applied voltage would simply be to generate H_2_ gas, which then participates in thermo-HFN at the catalyst site. If this were true, we would expect that at the same local CO and H_2_ concentrations, the electro-HFN rate should equal the thermo-HFN rate. However, we exclude this hypothesis because at similar reaction conditions, the rate of electro-HFN is significantly faster than that of thermo-HFN. For example, we observed that the maximum rate for thermo-HFN was over 10 times lower than that of electro-HFN with the same catalyst and solvent conditions (Figure 1d,e).

**Figure 3:**
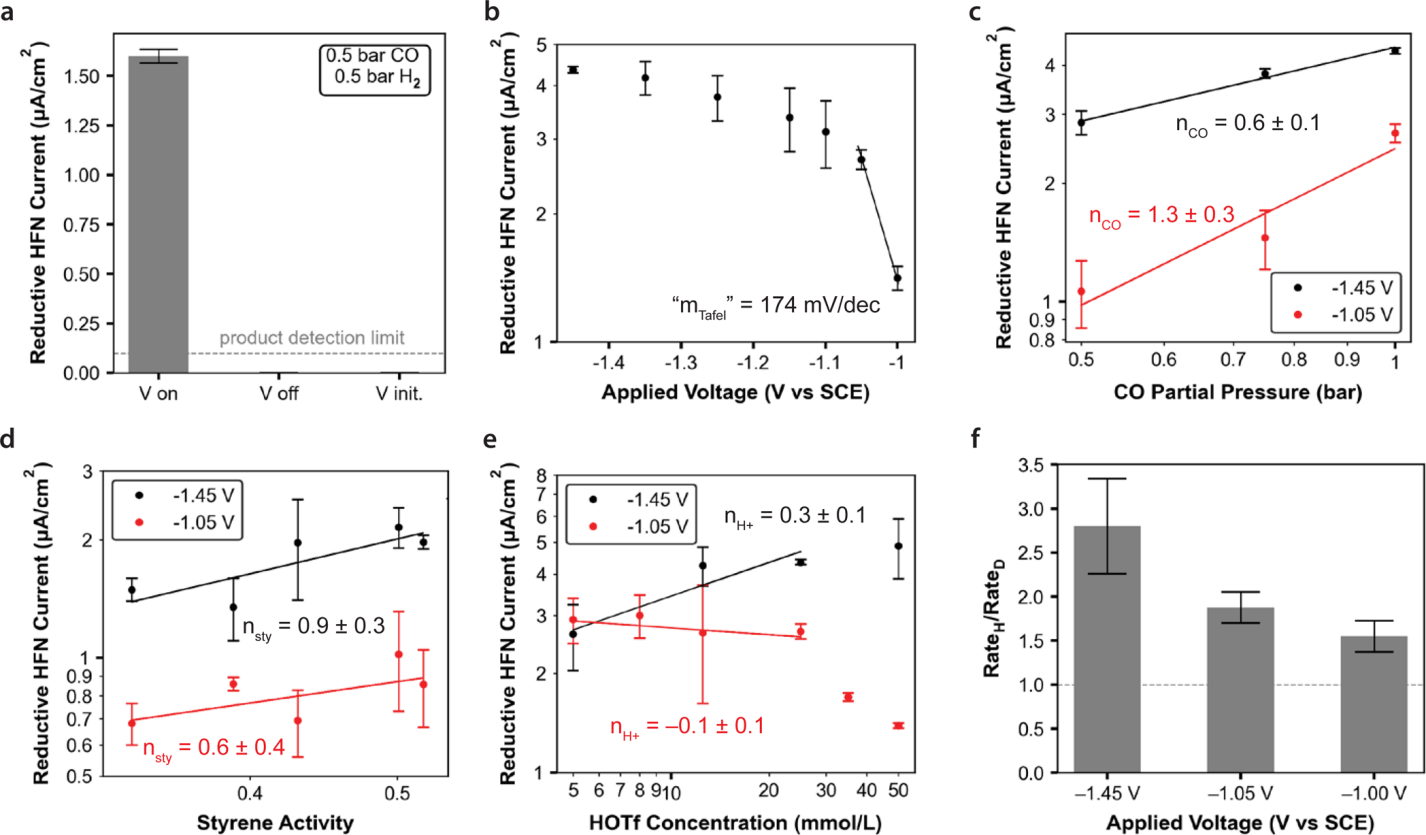
Rate data for electro-HFN at ambient temperature and pressure. (a) Control tests with and without application of voltage in the explicit presence of H_2_ gas. “V on”: constant application of −1.45 V vs SCE for the entire duration of the experiment, “V off”: no voltage applied, and “V init.”: −1.45 V vs SCE was applied for only the first 5 min of a 2 h experiment (b) Tafel dependence. (c) CO partial pressure dependence. (d) Styrene activity dependence. Data correspond to styrene concentrations ranging from 0.13 to 0.28 mol/L. (e) Acid concentration dependence. Panels (c–e) plotted on log–log scales, with data at higher driving forces (−1.45 V vs SCE) in black and data at lower driving forces (−1.05 V vs SCE) in red. (f) Kinetic isotope effect measurements, where the reaction was run with D_2_O, isopropanol-*d*_8_, and DOTf to replace possible proton sources with deuterium. Plotted is the rate with protons divided by the rate with deuterons. All data were collected at 25 °C, 1 bar CO, 0.52 M styrene, 25 mM HOTf, and 0.1 M TBAOTf in a 50% v/v IPA/H_2_O mixture unless explicitly labeled otherwise. Error bars represent standard deviation [propagated through division in (f)] with *n* ≥ 3.

A higher local concentration of H_2_ during electro-HFN is unlikely to account for this 10 times difference. First, mathematical analysis of H_2_ transport shows that similar local H_2_ concentrations are expected at the catalyst surfaces for the thermo- and electro-HFN conditions tested (Supporting Information Section 2.5). Even without this, from the apparent H_2_ saturation kinetics displayed in the thermo-HFN data (Figure 1d), any further increases in H_2_ pressure are unlikely to increase thermo-HFN rates. Finally, feeding 0.5 bar CO/0.5 bar H_2_ while applying voltage resulted in observable product, whereas flowing the same gas mixture with no applied voltage resulted in no observable product (Figure 3a).

The above differences could still be consistent with an indirect mechanism if applied voltage non-Faradaically promotes thermo-HFN. However, we did not find evidence of non-Faradaic promotion. We first tested whether applied voltage was only needed to activate the catalyst to a reactive state (e.g., help to generate surface hydrides necessary to enter a catalytic cycle) by feeding 0.5 bar CO/0.5 bar H_2_ and only applying voltage for the first 5 min of the reaction; we did not observe product (Figure 3a). Additionally, at elevated temperatures where the rate of thermo-HFN exceeded that of electro-HFN, we saw no evidence of non-Faradaic promotion upon the application of voltage (Figure S16 and Supporting Information Section 2.6).

### 2.5 Electrochemical Kinetic Analysis

To further probe the reaction mechanism, we collected electro-HFN reaction data via potentiostatic experiments at ambient pressure. We assumed steady-state rates, which we confirmed with time-dependent experiments (Figure S18). We also assumed transport-free kinetic control, which we confirmed with flow rate dependence experiments as well as a mathematical analysis indicating operation below 5% of the transport-limited current density (Supporting Information Section 2.8.1 and Figure S20).

The Tafel data show a weak voltage dependence that attenuates at more reductive potentials (Figure 3b). Fitting the points at the least reductive potentials yields a Tafel slope around 170 mV/dec, and fitting the most reductive potentials yields a slope greater than 1000 mV/dec. This saturation behavior is unlikely to be caused by mass transport limitations, as indicated via both experimental and theoretical mass transport analyses (*vide supra*). Instead, the Tafel curvature may arise from surface coverage effects.^27,28^

The CO order dependence data also show saturation behavior at more reductive potentials: the apparent CO order is linear (*n*_CO_ = 1.3 ± 0.3) at less reductive potentials but becomes sublinear (*n*_CO_ = 0.6 ± 0.1) at more reductive potentials (Figure 3c). Thus, the attenuation of both electron (Tafel) and CO apparent orders at more reductive potentials may be ascribed to the buildup of a surface intermediate (*I*_1_, Figure S28a) that is formed after these two species participate in the catalytic cycle. Notably, both positive^21^ and negative^15^ CO dependencies have been reported at similar heterogeneous thermo-HFN catalysts.

The styrene dependence is shown as a function of styrene activity, which was experimentally measured using headspace GC–MS measurements (Supporting Information Section 1.4.8). The styrene dependence, unlike that of electrons and CO, does not appear to be significantly potential-dependent. At both less reductive (*n*_sty_ = 0.6 ± 0.4) and more reductive (*n*_sty_ = 0.9 ± 0.3) potentials, the styrene dependences are within error of each other (Figure 3c). Interpreting these slopes to be within error of 1, these trends may be consistent with a mechanism where styrene enters the catalytic cycle after *I*_1_ is formed. However, these observations are also within error of other qualitative interpretations. Furthermore, various observations suggest that styrene may play several roles in the catalytic system, so these kinetic measurements may even reflect a convolution of several different effects. For example, the *operando* XANES data show that the presence of styrene influences the response of Rh valency to the applied potential (*vide supra*), potentially indicating a second-order influence of styrene on the catalyst itself. Additionally, above the solubility limit of styrene where phase separation begins to occur in the electrolyte, there is an apparent discontinuity in the styrene order dependence, where the rate sharply increases (Figure S21a). The reason why phase separation induces improved rates may be related to mass transport but remains to be determined.

From the proton rate data, the reaction appears to be proton independent (*n*_H+_ = −0.1 ± 0.1) at less reductive potentials and weakly proton-dependent (*n*_H+_ = 0.3 ± 0.1) at more reductive potentials (Figure 3e). Additionally, the magnitude of the KIE increases at more reductive potentials (Figure 3f), which is consistent with a proton or a proton-derived hydrogen atom becoming more kinetically relevant at more reductive potentials. However, at a lower styrene concentration, we observe apparent zero-order proton kinetics at both less and more reductive potentials (Figure S22c). These disparate observations remain to be fully reconciled but may be indications that the proton donor, HOTf, influences the reaction rate in a complex fashion beyond simply providing protons. For example, we observe that simply substituting HOTf with other strong acids such as phosphoric, nitric, or sulfuric acid, even while maintaining TBAOTf as the supporting electrolyte, suppresses reactivity. Thus, these conflicting observations, coupled with the large variability in these kinetic data, do not currently provide a clear indication of how protons participate in the reaction mechanism.

The above Tafel, CO, and proton-dependent measurements were taken at 0.52 M styrene, which is representative of the optimal operating condition but is also above the styrene solubility limit. Kinetic data taken in single-phase electrolyte solutions with 0.28 M styrene are provided in Figure S22. Additionally, reaction rate data for hydrogenation, HER, and total current corresponding to Figure 3b–e are presented in Figures S22–24.

### 2.6 Summary of Mechanistic Interpretations

The preceding experimental observations, taken all together, provide several insights about the mechanism of electro-HFN.

First, comparison of thermo- and electro-HFN rates (Figure 1d,e) strongly suggests that electro-HFN proceeds through a mechanism that is microscopically distinct from that of thermo-HFN. Specifically, we expect that protons and electrons directly participate in the catalytic cycle and do not simply serve to form H_2_*in situ*.

Second, Rh loading dependence tests (Figure S15a) suggest that unlike electro-HFN, a significant amount of the side reactions (hydrogenation, HER) may be attributed to non-Rh sites. This may be useful for guiding selectivity improvements in future systems because the principal side reactions are likely associated with different sites than those of electro-HFN.

Finally, the mechanism of electro-HFN appears to be complex, and variability in the kinetic data enables only a speculative mechanistic interpretation. The data support a mechanism that involves the buildup of some surface intermediate (*I*_1_) at more reductive potentials. Potential-dependent saturation behavior in both the Tafel and CO dependence data suggest that electrons and CO participate in the catalytic cycle before *I*_1_ forms. While the data do not provide a strong indication of the chemical identity of *I*_1_, several speculative chemical interpretations are provided in Figure S28. The styrene and proton dependencies do not display the same voltage-dependent attenuation, perhaps suggesting that these species do not participate in the mechanism before *I*_1_ forms. However, due to the greater variability in the proton and styrene order dependence data, as well as the possible second-order roles of these reactants, it is less clear how these species participate in the mechanism. Their roles, as well as further elucidation of the electro-HFN mechanism, remain to be determined from future studies. Further mechanistic discussion is provided in Supporting Information Section 2.12.

### 2.7 Extension of the Reactor and Substrate Scope

**Figure 4:**
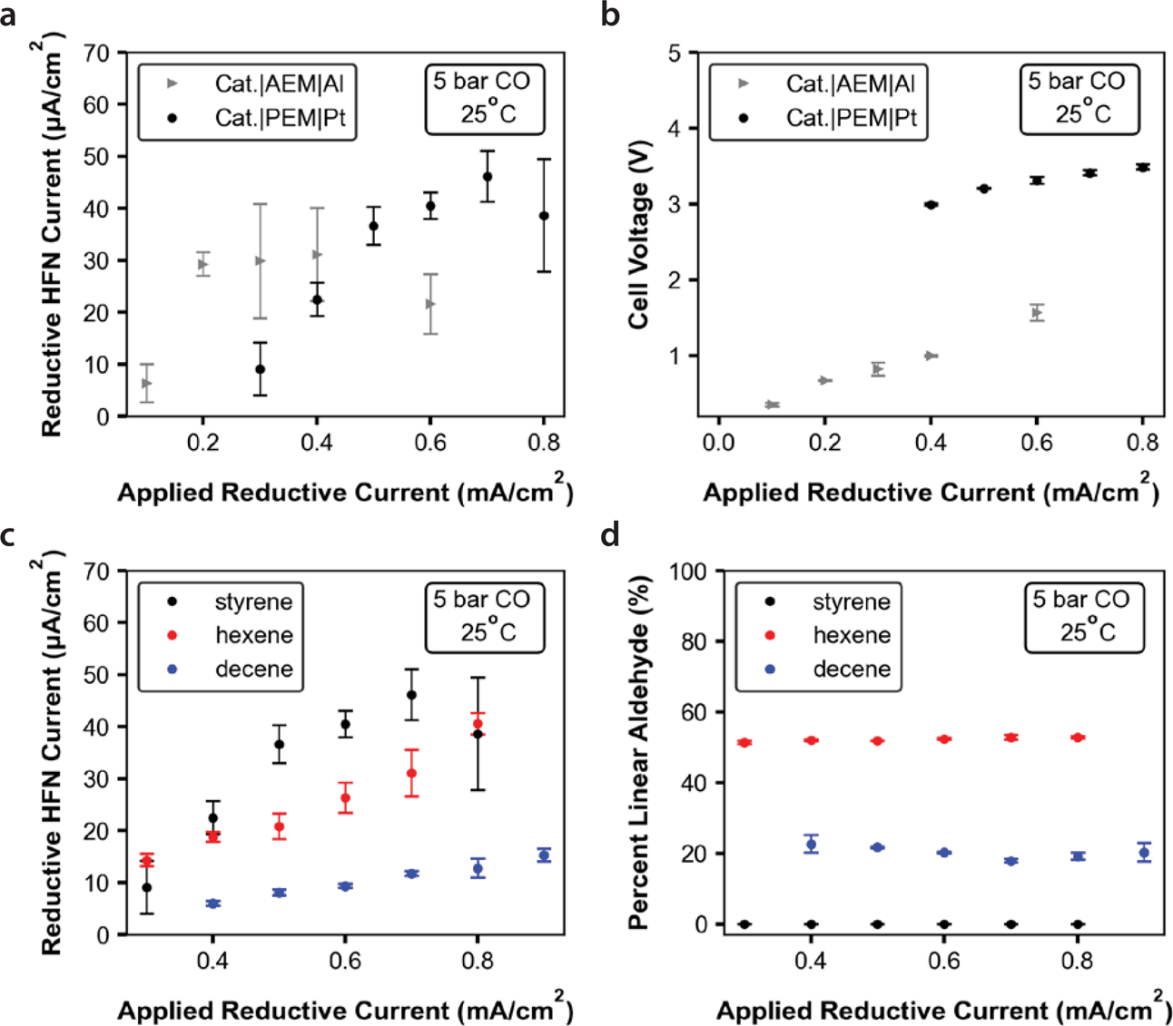
Extension of the reactor and substrate scope. (a) Electro-HFN TOF as a function of applied reductive current for cells containing either a sacrificial Al anode and AEM (gray) or a Pt anode and a cation exchange membrane (black). (b) Cell voltages corresponding to the reaction rate data shown in (a). (c) Electro-HFN TOF as a function of applied reductive current for styrene (black), 1-hexene (red), and 1-decene (blue). (d) Electro-HFN regioselectivity for the same three olefins, plotted as a percentage of the aldehyde products with linear selectivity. Unless labeled otherwise, all data were collected beyond the saturation limit of the olefin and at 25 °C, 5 bar CO, 0.1 M TBAOTf, 25 mM HOTf in a 50% v/v IPA/H_2_O mixture, with a Pt anode and Nafion membrane. Error bars represent standard deviation with *n* ≥ 3.

Finally, we investigated strategies to improve the practical relevance of our reported electro-HFN chemistry. First, we replaced the aluminum sacrificial anode with a Pt anode so that water oxidation, rather than aluminum oxidation, could serve as the anodic counter reaction and generate protons to replenish those consumed by electro-HFN. We also replaced the anion exchange membrane (AEM) with a proton exchange (Nafion) membrane. In this new cell configuration, the overall reaction is hydroformylation with water as the cheap and abundant hydrogen atom source. For galvanostatic experiments at 5 bar CO and 25 °C, this new reaction setup achieves a higher maximum current for electro-HFN (45 μA/cm^2^) than that of the original reactor configuration (30 μA/cm^2^) (Figure 4a). We note that using water, rather than aluminum, oxidation at the anode incurs a cell voltage penalty of about 2 V (Figure 4b).

Additionally, we extended the substrate scope to include 1-hexene and 1-decene, which are chemically representative of the linear, nonaromatic olefins that are principally used in industrial hydroformylation processes. For galvanostatic experiments at 5 bar CO, 25 °C, and above the saturation limit of the olefin, the rates and Faradaic efficiencies for 1-hexene were almost comparable to those for styrene, and those for 1-decene were slightly lower. Maximum partial currents for electro-HFN were ca. 45, 40, and 15 μA/cm^2^ for styrene, 1-hexene, and 1-decene, respectively (Figure 4c). Unlike styrene, where only the branched product was observed, both the linear and branched aldehyde products were observed for 1-hexene (ca. 50% linear) and 1-decene (ca. 20% linear) (Figure 4d).

## 3 Conclusions

Our results demonstrate that a single catalyst composition can be competent for both thermo- and electro-HFN, in contrast to what has previously been observed.^13^ This insight is important because it allowed us to use known thermochemical reactivity as a direct experimental starting point for designing a new electrocatalyst. Inspiration from industrial hydrotreating catalysts has helped inspire material choices for hydrogen evolution electrocatalysts,^29^ and catalysts competent for both the thermocatalytic and electrocatalytic versions of the same reaction have been reported in many contexts including CO_2_ hydrogenation,^30^ hydrogen oxidation,^31^ CO oxidation,^32^ oxygen reduction,^33−36^ hydrogenation,^37^ and nitrate reduction.^38^ However, these efforts are limited to oxidation or reduction of small molecules, and typically, both the thermochemical and electrochemical reactions are already independently well-studied. Our work adds to this by demonstrating how the thermocatalysis and electrocatalysis divide can be experimentally traversed to expose even more complex reactivity such as C–C bond formation.

Importantly, we also show that electro-HFN is mechanistically distinct from thermo-HFN. Therefore, our electrified hydroformylation analogue is fundamentally different from previously reported electrified analogues of other organometallic thermochemistries^39^ such as oxidative C–H activation^40^ and reductive cross-coupling.^41,42^ In these systems, catalysis occurs at a homogeneous site, and voltage indirectly participates in catalysis. Specifically, voltage replaces a sacrificial reductant or oxidant, and its role is to perform outer sphere electron transfer to generate an active species (such as regenerating an active Ni species)^41,42^ that then thermochemically participates in known catalysis. Thus, there was minimal precedent to observe a change in reaction mechanism upon electrifying a hydroformylation catalyst.

This mechanistically distinct and directly voltage-driven pathway has several important implications. First, from a practical perspective, we show that accessing electrochemical pathways lead to improved reactivity at ambient temperature. Thermo-HFN is typically performed at elevated temperatures (90–120 °C), and the voltage-driven pathway may enable operation at milder temperatures. For a comparison of rates in the broader literature, heterogeneous thermo-HFN rates typically range from 100 to 3000 h^–1^. However, these rates are not directly comparable to our observed electro-HFN rates (up to ca. 1 h^–1^) because they require elevated temperatures ranging from 60 to 150 °C. Rates at room temperature are not reported presumably because the catalysis is much more sluggish.^14,15,21,43,44^ The electro-HFN rates we observe are not yet competitive with those reported for ambient temperature homogeneous thermo-HFN (10–25 h^–1^),^45,46^ but achieving parity between heterogeneous and homogeneous systems is a general challenge in hydroformylation chemistry.

Second, because thermo-HFN with H_2_ gas is exergonic, the equilibrium potential of electro-HFN occurs at a less reductive potential than that of HER. Thus, a direct electro-HFN reaction could be more energy efficient than a thermo-HFN process that uses HER-derived H_2_. However, we note that the voltages reported in this work are still ∼1 V more reductive than those required for HER (Supporting Information Section 2.13), so further reaction optimization will be a subject of future work.

Finally, the microscopically distinct reaction mechanisms of thermo- vs electro-HFN may also allow for unique catalyst design principles. For example, because electro-HFN does not proceed through H_2_ as an intermediate, an electro-HFN catalyst does not need to catalytically activate H_2_ to generate hydrides; it simply needs to accept protons and electrons to do so. Thus, electro-HFN catalysts could be further optimized for earlier steps in the hydroformylation cycle such as olefin activation or C–C bond formation without being constrained by the need to activate H_2_. Additionally, we observed that in some reaction conditions, Rh valency changed with applied potential. This suggests that voltage might also be useful as a handle to change the catalyst electronic structure in addition to those of ligand and support identity that are typically used in thermocatalysis.

Looking forward, while the performance of this initial electro-HFN demonstration remains modest, strategies for further improvements include exploring new catalysts, electrode or electrolyte formulations, and cell designs. For example, since much of the side reactivity occurs even without the Rh-based catalyst present, it may be possible to improve selectivity by altering the electrode composition. Additionally, since the volume fraction of water, as well as the presence of triflate salts, had significant effects on reaction rates, continued exploration of electrolyte composition may further improve performance. As rates improve, reactor development for improved mass transport of gas-phase reactants will likely be important as well.

To conclude, this work establishes an experimental strategy for directly electrifying promising thermocatalysts. This strategy allows for the development of new voltage-driven reactions that simultaneously improve upon known thermochemical reactivity and expand the electrochemical reaction toolkit.

## 4 Methods

### 4.1 Catalyst and Electrode Preparation

CeO_2_ nanoparticle synthesis was adapted from the literature.^14^ Briefly, cerium(III) nitrate hexahydrate (Beantown Chemical, 99.99%) was ground with a mortar and pestle, put in a combustion boat, and calcined under nitrogen flow at 350 °C for 2 h in a tube furnace. The resulting light-yellow solid was ground again with a mortar and pestle.

Rh@CeO_2_ was synthesized via a wetness impregnation procedure that was adapted from the literature.^14^ In summary, 1 g of CeO_2_ powder was combined with 99 mg of rhodium(III) acetylacetylonate (Sigma-Aldrich, 97%) and approximately 3 mL of acetone. The mixture was ground with a mortar and pestle until all of the acetone evaporated. The resulting powder was dried at 80 °C for 30 min and then heated in static air in a muffle furnace at 800 °C for 10 h with a 10 °C/min ramp rate.

Rh@CeO_2_ electrodes were prepared by drop casting catalyst ink solutions [Rh@CeO_2_, Nafion 117 solution (Sigma-Aldrich, 5%) and carbon black (Vulcan XC 72, Fuel Cell Store) in isopropanol] onto hydrophilic carbon paper. The catalyst loading was kept at 3.77 mg Rh@CeO_2_ per cm^2^ electrode. Electrodes were dried at 80 °C for 30 min and then annealed at 150 °C for 6 h.

### 4.2 Electrolyte Preparation and Characterization

The default electrolyte mixture used in these studies was a 50/50 v/v % water/isopropanol mixture with 0.1 M tetrabutylammonium trifluoromethanesulfonate (Sigma-Aldrich, 99%) and 0.025 M trifluoromethanesulfonic acid (Sigma-Aldrich, 99%). Directly prior to performing a reaction, this electrolyte was combined with styrene (Sigma-Aldrich, 99%) to afford 0.52 M styrene in the electrolyte solution. Notably, 0.52 M styrene is above the solubility limit, so the final electrolyte was cloudy with a small amount of phase separation. For styrene order dependence studies, the activity of styrene was experimentally measured using previously reported headspace GC methods.^47^

### 4.3 Thermochemical Reaction Setup

Room-temperature thermochemical reactions were performed as 2 mL scale batch reactions within Parr reactors. These reactions were performed such that parameters such as catalyst loading/preparation, electrolyte, reactant concentration, etc., were directly comparable to electrochemical reactions. Reactions were typically run for 14–18 h.

### 4.4 Electrochemical Reaction Setup

Electrochemical measurements were performed in PEEK sandwich cells in a two-compartment configuration. Aluminum foil (Reynold’s Wrap) was used as the sacrificial counter electrode, Neosepta AHA membranes (Ameridia Innovative Solutions) were used as the separator, and when applicable, a leak-free Ag/AgCl electrode (Innovative Instruments LF-2) was used as the reference electrode. Anode and cathode compartments were filled with 2 mL of electrolyte solution each. A magnetic stir bar was also added to the cathode compartment, and the cell was stirred at 700 rpm during the reaction.

Elevated pressure experiments were typically performed with a static, pressurized gas headspace, whereas ambient pressure experiments were performed with 10 sccm of CO gas bubbling through the catholyte.

Application of potential was performed using a potentiostat (Biologic VMP-3e). CA measurements were run with 90% automatic resistance compensation, where electrolyte resistance was determined using potentiostatic electrochemical impedance spectroscopy measurements. Typical electrochemical experiments, at both elevated and ambient pressures, were run for 1–3 h.

### 4.5 Reaction Workup and Product Quantification

For product quantification, 2 mL of sample liquid was collected. For room-temperature thermochemical measurements, this corresponded to the entire reaction mixture, and for electrochemical measurements, this corresponded to the catholyte only (product crossover to the anolyte was confirmed to be negligible). To the reaction liquid, 20 μL of 0.16 M 1,3,5-trimethoxybenzene (Sigma 138827) solution in IPA was added as an internal standard. Then, 1 mL of acetonitrile and 1 mL of Milli-Q water were added. Finally, the reaction mixture was extracted three times using 500 μL of hexanes for each extraction. A gas chromatograph–mass spectrometer (7890B GC, Agilent) fitted with a DB-WAX column and a flame ionization detector were used to identify and quantify products. Calibration curves were constructed with known standards to quantify both the extraction efficiency and GC-FID and GCMS response factor.

### 4.6 XAS Measurements

Both *operando* and *ex situ* XAS measurements were performed at the Inner Shell Spectroscopy (ISS) beamline^22^ at the Brookhaven National Laboratory. ISS is a damping wiggler beamline with an energy range of 4.9–33 keV and a flux of 5 × 10^13^ photons/s at 12 keV. Catalyst preparation for the XAS studies was identical to that for the reactivity studies. Additionally, XAS measurements were taken using a standard electrochemical cell, with the only modification being the incorporation of a Kapton (X-ray transparent) window to the working electrode cover plate (Figure S1). Thus, the *operando* XAS measurements were expected to have identical reactor transport properties to those of the reactivity measurements performed at ambient pressure. Reference materials employed for these studies were Rh_2_O_3_ powder (Sigma-Aldrich 204226) and Rh(0) foil.

All XAS measurements on catalyst samples were collected in fluorescence mode. For any set of electrolyte/gas conditions, data for all voltages were collected sequentially from the least reductive to most reductive potential. At each potential, the sample was allowed to reach steady state for 10 min, and then 30 scans were collected at different locations on the electrode, where different locations were autodefined by a roughly square-shaped grid pattern with 1 mm spacing between locations. The beam spot size was 1 × 1 mm (fwhm). For different electrolyte/gas settings, the cell was disassembled, cleaned, and prepared with a fresh electrode and electrolyte.

## Supporting Information Available

Reagent information, detailed experimental protocols, additional data, and additional discussion (PDF)

## Abbreviations

Electro-HFN: electrochemical hydroformylation
EXAFS: extended X-ray absorption fine structure
ET: electron transfer
FE: Faradaic efficiency
PT: proton transfer
Thermo-HFN: thermochemical hydroformylation
TOF: turnover frequency
XANES: X-ray absorption near edge spectroscopy
XAS: X-ray absorption spectroscopy

## References

[ref1] Schiffer Z. J.; Manthiram K. Electrification and Decarbonization of the Chemical Industry. Joule 2017, 1 (1), 10–14. 10.1016/j.joule.2017.07.008.

[ref2] Xia R.; Overa S.; Jiao F. Emerging Electrochemical Processes to Decarbonize the Chemical Industry. JACS Au 2022, 2 (5), 1054–1070. 10.1021/jacsau.2c00138. 35647596 PMC9131369

[ref3] Mallapragada D. S.; Dvorkin Y.; Modestino M. A.; Esposito D. V.; Smith W. A.; Hodge B. M.; Harold M. P.; Donnelly V. M.; Nuz A.; Bloomquist C.; Baker K.; Grabow L. C.; Yan Y.; Rajput N. N.; Hartman R. L.; Biddinger E. J.; Aydil E. S.; Taylor A. D. Decarbonization of the Chemical Industry through Electrification: Barriers and Opportunities. Joule 2023, 7 (1), 23–41. 10.1016/j.joule.2022.12.008.

[ref4] Weissermel K.; Arpe H.-J. Industrial Organic Chemistry, 4th ed.; WILEY-VCH Verlag GmbH & Co. KGaA, 2003.

[ref5] Franke R.; Selent D.; Börner A. Applied Hydroformylation. Chem. Rev. 2012, 112 (11), 5675–5732. 10.1021/cr3001803. 22937803

[ref6] Zhang B.; Peña Fuentes D.; Börner A. Hydroformylation. ChemTexts 2022, 8 (1), 2 10.1007/s40828-021-00154-x.

[ref7] Kohlpaintner C.; Schulte M.; Falbe J.; Lappe P.; Weber J.; Frey G. D. Aldehydes, Aliphatic. In Ullmann’s Encyclopedia of Industrial Chemistry; Wiley-VCH Verlag GmbH & Co., 2013. 10.1002/14356007.A01_321.PUB3.

[ref8] Birdja Y. Y.; Pérez-Gallent E.; Figueiredo M. C.; Göttle A. J.; Calle-Vallejo F.; Koper M. T. M. Advances and Challenges in Understanding the Electrocatalytic Conversion of Carbon Dioxide to Fuels. Nat. Energy 2019, 4 (9), 732–745. 10.1038/s41560-019-0450-y.

[ref9] Zhuang T.-T.; Pang Y.; Liang Z.-Q.; Wang Z.; Li Y.; Tan C.-S.; Li J.; Dinh C. T.; De Luna P.; Hsieh P.-L.; Burdyny T.; Li H.-H.; Liu M.; Wang Y.; Li F.; Proppe A.; Johnston A.; Nam D.-H.; Wu Z.-Y.; Zheng Y.-R.; Ip A. H.; Tan H.; Chen L.-J.; Yu S.-H.; Kelley S. O.; Sinton D.; Sargent E. H. Copper Nanocavities Confine Intermediates for Efficient Electrosynthesis of C3 Alcohol Fuels from Carbon Monoxide. Nat. Catal. 2018, 1 (12), 946–951. 10.1038/s41929-018-0168-4.

[ref10] Ren D.; Wong N. T.; Handoko A. D.; Huang Y.; Yeo B. S. Mechanistic Insights into the Enhanced Activity and Stability of Agglomerated Cu Nanocrystals for the Electrochemical Reduction of Carbon Dioxide to N-Propanol. J. Phys. Chem. Lett. 2016, 7 (1), 20–24. 10.1021/acs.jpclett.5b02554. 26740140

[ref11] Resasco J.; Bell A. T. Electrocatalytic CO_2_ Reduction to Fuels: Progress and Opportunities. Trends Chem. 2020, 2 (9), 825–836. 10.1016/j.trechm.2020.06.007.

[ref12] Pan F.; Yang Y. Designing CO_2_ Reduction Electrode Materials by Morphology and Interface Engineering. Energy Environ. Sci. 2020, 13 (8), 2275–2309. 10.1039/D0EE00900H.

[ref13] Otsuka K.; Ando T.; Yamanaka I. Hydroformylation of Ethylene via Spontaneous Cell Reactions in the Gas Phase. J. Catal. 1997, 165 (2), 221–230. 10.1006/jcat.1997.1472.

[ref14] Amsler J.; Sarma B. B.; Agostini G.; Prieto G.; Plessow P. N.; Studt F. Prospects of Heterogeneous Hydroformylation with Supported Single Atom Catalysts. J. Am. Chem. Soc. 2020, 142 (11), 5087–5096. 10.1021/jacs.9b12171. 32141745

[ref15] Ro I.; Xu M.; Graham G. W.; Pan X.; Christopher P. Synthesis of Heteroatom Rh–ReO_x_ Atomically Dispersed Species on Al_2_O_3_ and Their Tunable Catalytic Reactivity in Ethylene Hydroformylation. ACS Catal. 2019, 9 (12), 10899–10912. 10.1021/acscatal.9b02111.

[ref16] Tuller H. L.; Nowick A. S. Defect Structure and Electrical Properties of Nonstoichiometric CeO_2_ Single Crystals. J. Electrochem. Soc. 1979, 126 (2), 209–217. 10.1149/1.2129007.

[ref17] Chavhan M. P.; Som S.; Lu C.-H. Size-Controlled Ceria Nanocubes Obtained via Hydrothermal Route for Electrochemical Capacitors. Mater. Lett. 2019, 257, 126598 10.1016/j.matlet.2019.126598.

[ref18] Samantaray Y.; Martin D. J.; Agarwal R. G.; Gibson N. J.; Mayer J. M. Proton-Coupled Electron Transfer of Cerium Oxide Nanoparticle Thin-Film Electrodes. J. Phys. Chem. C 2023, 127 (8), 4015–4020. 10.1021/acs.jpcc.2c06783.

[ref19] Lazzaroni R.; Raffaelli A.; Settambolo R.; Bertozzi S.; Vitulli G. Regioselectivity in the Rhodium-Catalyzed Hydroformylation of Styrene as a Function of Reaction Temperature and Gas Pressure. J. Mol. Catal. 1989, 50 (1), 1–9. 10.1016/0304-5102(89)80104-X.

[ref20] Zhao J.; He Y.; Wang F.; Zheng W.; Huo C.; Liu X.; Jiao H.; Yang Y.; Li Y.; Wen X. Suppressing Metal Leaching in a Supported Co/SiO_2_ Catalyst with Effective Protectants in the Hydroformylation Reaction. ACS Catal. 2020, 10 (2), 914–920. 10.1021/acscatal.9b03228.

[ref21] Ro I.; Qi J.; Lee S.; Xu M.; Yan X.; Xie Z.; Zakem G.; Morales A.; Chen J. G.; Pan X.; Vlachos D. G.; Caratzoulas S.; Christopher P. Bifunctional Hydroformylation on Heterogeneous Rh-WOx Pair Site Catalysts. Nature 2022, 609 (7926), 287–292. 10.1038/s41586-022-05075-4. 36071187

[ref22] Leshchev D.; Rakitin M.; Luvizotto B.; Kadyrov R.; Ravel B.; Attenkofer K.; Stavitski E. The Inner Shell Spectroscopy Beamline at NSLS-II: A Facility for in Situ and Operando X-Ray Absorption Spectroscopy for Materials Research. J. Synchrotron Radiat. 2022, 29 (4), 1095–1106. 10.1107/s160057752200460x. 35787577 PMC9255565

[ref23] Ravel B.; Newville M. ATHENA, ARTEMIS, HEPHAESTUS: Data Analysis for X-Ray Absorption Spectroscopy Using IFEFFIT. J. Synchrotron Radiat. 2005, 12 (4), 537–541. 10.1107/S0909049505012719. 15968136

[ref24] Matsubu J. C.; Yang V. N.; Christopher P. Isolated Metal Active Site Concentration and Stability Control Catalytic CO_2_ Reduction Selectivity. J. Am. Chem. Soc. 2015, 137 (8), 3076–3084. 10.1021/ja5128133. 25671686

[ref25] Farpón M. G.; Henao W.; Plessow P. N.; Andrés E.; Arenal R.; Marini C.; Agostini G.; Studt F.; Prieto G. Rhodium Single-Atom Catalyst Design through Oxide Support Modulation for Selective Gas-Phase Ethylene Hydroformylation. Angew. Chem., Int. Ed. 2023, 62 (1), e202214048 10.1002/anie.202214048. PMC10099584 36315420

[ref26] Yu Z.; Zhang S.; Zhang L.; Liu X.; Jia Z.; Li L.; Ta N.; Wang A.; Liu W.; Wang A.; Zhang T. Suppressing Metal Leaching and Sintering in Hydroformylation Reaction by Modulating the Coordination of Rh Single Atoms with Reactants. J. Am. Chem. Soc. 2024, 146 (17), 11955–11967. 10.1021/jacs.4c01315. 38640231

[ref27] Zeng J. S.; Corbin N.; Williams K.; Manthiram K. Kinetic Analysis on the Role of Bicarbonate in Carbon Dioxide Electroreduction at Immobilized Cobalt Phthalocyanine. ACS Catal. 2020, 10 (7), 4326–4336. 10.1021/acscatal.9b05272.

[ref28] Shinagawa T.; Garcia-Esparza A. T.; Takanabe K. Insight on Tafel Slopes from a Microkinetic Analysis of Aqueous Electrocatalysis for Energy Conversion. Sci. Rep. 2015, 5 (1), 13801 10.1038/srep13801. 26348156 PMC4642571

[ref29] Morales-Guio C. G.; Stern L. A.; Hu X. Nanostructured Hydrotreating Catalysts for Electrochemical Hydrogen Evolution. Chem. Soc. Rev. 2014, 43 (18), 6555–6569. 10.1039/C3CS60468C. 24626338

[ref30] Koshy D. M.; Nathan S. S.; Asundi A. S.; Abdellah A. M.; Dull S. M.; Cullen D. A.; Higgins D.; Bao Z.; Bent S. F.; Jaramillo T. F. Bridging Thermal Catalysis and Electrocatalysis: Catalyzing CO_2_ Conversion with Carbon-Based Materials. Angew. Chem., Int. Ed. 2021, 60 (32), 17472–17480. 10.1002/anie.202101326. 33823079

[ref31] Stonehart P.; Ross P. N. The Commonality of Surface Processes in Electrocatalysis and Gas-Phase Heterogeneous Catalysis. Catal. Rev. 1975, 12 (1), 1–35. 10.1080/01614947508067520.

[ref32] Blurton K. F.; Stetter J. R. The Gas Phase and Electrochemical Oxidation of Carbon Monoxide on Platinum, Palladium and Ruthenium Catalysts: A Comparative Study. J. Catal. 1977, 46 (2), 230–233. 10.1016/0021-9517(77)90203-2.

[ref33] Bates J. S.; Biswas S.; Suh S.-E.; Johnson M. R.; Mondal B.; Root T. W.; Stahl S. S. Chemical and Electrochemical O_2_ Reduction on Earth-Abundant M-N-C Catalysts and Implications for Mediated Electrolysis. J. Am. Chem. Soc. 2022, 144 (2), 922–927. 10.1021/jacs.1c11126. 34985869 PMC8833842

[ref34] Adams J. S.; Kromer M. L.; Rodríguez-López J.; Flaherty D. W. Unifying Concepts in Electro- And Thermocatalysis toward Hydrogen Peroxide Production. J. Am. Chem. Soc. 2021, 143 (21), 7940–7957. 10.1021/jacs.0c13399. 34019397

[ref35] Ryu J.; Bregante D. T.; Howland W. C.; Bisbey R. P.; Kaminsky C. J.; Surendranath Y. Thermochemical Aerobic Oxidation Catalysis in Water Can Be Analysed as Two Coupled Electrochemical Half-Reactions. Nat. Catal. 2021, 4 (9), 742–752. 10.1038/s41929-021-00666-2.

[ref36] Howland W. C.; Gerken J. B.; Stahl S. S.; Surendranath Y. Thermal Hydroquinone Oxidation on Co/N-Doped Carbon Proceeds by a Band-Mediated Electrochemical Mechanism. J. Am. Chem. Soc. 2022, 144 (25), 11253–11262. 10.1021/jacs.2c02746. 35699525

[ref37] Singh N.; Sanyal U.; Ruehl G.; Stoerzinger K. A.; Gutiérrez O. Y.; Camaioni D. M.; Fulton J. L.; Lercher J. A.; Campbell C. T. Aqueous Phase Catalytic and Electrocatalytic Hydrogenation of Phenol and Benzaldehyde over Platinum Group Metals. J. Catal. 2020, 382, 372–384. 10.1016/j.jcat.2019.12.034.

[ref38] Wang Z.; Ortiz E. M.; Goldsmith B. R.; Singh N. Comparing Electrocatalytic and Thermocatalytic Conversion of Nitrate on Platinum-Ruthenium Alloys. Catal. Sci. Technol. 2021, 11 (21), 7098–7109. 10.1039/D1CY01075A.

[ref39] Liu J.; Lu L.; Wood D.; Lin S. New Redox Strategies in Organic Synthesis by Means of Electrochemistry and Photochemistry. ACS Cent. Sci. 2020, 6 (8), 1317–1340. 10.1021/acscentsci.0c00549. 32875074 PMC7453421

[ref40] Shrestha A.; Lee M.; Dunn A. L.; Sanford M. S. Palladium-Catalyzed C-H Bond Acetoxylation via Electrochemical Oxidation. Org. Lett. 2018, 20 (1), 204–207. 10.1021/acs.orglett.7b03559. 29272130 PMC5772685

[ref41] DeLano T. J.; Reisman S. E. Enantioselective Electroreductive Coupling of Alkenyl and Benzyl Halides via Nickel Catalysis. ACS Catal. 2019, 9 (8), 6751–6754. 10.1021/acscatal.9b01785. 32351776 PMC7190267

[ref42] Perkins R. J.; Hughes A. J.; Weix D. J.; Hansen E. C. Metal-Reductant-Free Electrochemical Nickel-Catalyzed Couplings of Aryl and Alkyl Bromides in Acetonitrile. Org. Process Res. Dev. 2019, 23 (8), 1746–1751. 10.1021/acs.oprd.9b00232.

[ref43] Gorbunov D.; Safronova D.; Kardasheva Y.; Maximov A.; Rosenberg E.; Karakhanov E. New Heterogeneous Rh-Containing Catalysts Immobilized on a Hybrid Organic-Inorganic Surface for Hydroformylation of Unsaturated Compounds. ACS Appl. Mater. Interfaces 2018, 10 (31), 26566–26575. 10.1021/acsami.8b02797. 29979868

[ref44] Liu B.; Huang N.; Wang Y.; Lan X.; Wang T. Promotion of Inorganic Phosphorus on Rh Catalysts in Styrene Hydroformylation: Geometric and Electronic Effects. ACS Catal. 2021, 11 (3), 1787–1796. 10.1021/acscatal.0c04684.

[ref45] Brown C. K.; Wilkinson G. Homogeneous Hydroformylation of Alkenes with Hydridocarbonyltris-(Triphenylphosphine)Rhodium(I) as Catalyst. J. Chem. Soc. A 1970, 2753–2764. 10.1039/j19700002753.

[ref46] Seiche W.; Schuschkowski A.; Breit B. Bidentate Ligands by Self-Assembly through Hydrogen Bonding: A General Room Temperature/Ambient Pressure Regioselective Hydroformylation of Terminal Alkenes. Adv. Synth. Catal. 2005, 347 (11–13), 1488–1494. 10.1002/adsc.200505174.

[ref47] Williams K.; Limaye A.; Weiss T.; Chung M.; Manthiram K. Accounting for Species’ Thermodynamic Activities Changes Mechanistic Interpretations of Electrochemical Kinetic Data. 2022, ChemRxiv https://doi.org/10.26434/chemrxiv-2022-vk5z9 (accessed November 01, 2022).

