# Supplementary material for: Electrifying Hydroformylation Catalysts Exposes Voltage-Driven C–C Bond Formation
Source: J Am Chem Soc. 2024 Jun 10;146(24):16521–30. doi: 10.1021/jacs.4c02992 (PMC11191585; doi:10.1021/jacs.4c02992)
Supplement: Supplementary file 1 — ja4c02992_si_001.pdf [file ja4c02992_si_001.pdf]

**Electrifying hydroformylation catalysts exposes voltage-driven C–C bond formation**

Joy S. Zeng,[1] Emma L. Cosner,[2†] Spencer P. Delgado-Kukuczka,[2†] Chenyu Jiang,[2] Jason S. Adams,[2] Yuriy Román-Leshkov,[1] Karthish Manthiram[2,*]

[1]Department of Chemical Engineering, Massachusetts Institute of Technology, 77 Massachusetts Avenue, Cambridge, Massachusetts 02139, United States

[2]Division of Chemistry and Chemical Engineering, California Institute of Technology, Pasadena, California 91125, United States

[†]Equal contribution

*Correspondence: karthish@caltech.edu

# Table of Contents

1. Materials and Methods ..... 3
1.1 Materials List ..... 3
1.2 Catalyst and Electrode Preparation ..... 3
1.2.1 $CeO_2$ nanoparticle synthesis ..... 3
1.2.2 Rh@$CeO_2$ synthesis ..... 3
1.2.3 Carbon paper support preparation ..... 4
1.2.4 Working electrode (Rh@$CeO_2$ on carbon paper) preparation ..... 4
1.3 Catalyst Characterizations ..... 4
1.3.1 Quantifying Rh loading for Rh@$CeO_2$ particles via ICP-MS ..... 4
1.3.2 Powder X-ray diffraction measurements ..... 4
1.3.3 XPS measurements ..... 5
1.3.4 Synchrotron XAS measurements (*ex situ* and *operando*) ..... 5
1.3.5 XANES and EXAFS data analysis ..... 6
1.4 Reactivity Studies ..... 7
1.4.1 Electrolyte preparation ..... 7
1.4.2 Thermochemical reaction setup ..... 7
1.4.3 Electrochemical reaction setup ..... 7
1.4.4 Reactivity studies at elevated temperatures ..... 10
1.4.5 Reaction workup and quantification of liquid phase products ..... 10
1.4.6 Quantification of hydrogen gas evolution ..... 11
1.4.7 Calculation of turnover frequency and faradaic efficiency ..... 11
1.4.8 Experimental measurements of styrene and proton activity ..... 11
2. Supplemental data and discussion ..... 13

2.1 Supplemental catalyst characterization .......... 13
2.1.1 Transmission electron microscopy .......... 13
2.1.2 X-ray photoelectron spectroscopy .......... 13
2.1.3 Powder X-ray diffraction .......... 15
2.2 Characterizations during sequential electrification and reaction optimization .......... 15
2.2.1 Screening heterogenized Rh catalysts .......... 15
2.2.2 Finding ionically conductive media .......... 16
2.2.3 Electro- vs thermo- HFN at elevated temperatures .......... 16
2.2.4 Optimizing water content for electro-HFN at ambient temperature .......... 18
2.3 Faradaic Efficiency closure .......... 18
2.4 Control experiments on the nature of the electro-HFN site during catalysis .......... 20
2.5 Transport analysis of maximum local $H_2$ accumulation .......... 21
2.6 No evidence for non-Faradaic promotion .......... 22
2.7 Supplemental XAS data .......... 24
2.8 Control tests on the robustness of electrochemical rate data .......... 25
2.8.1 Analysis of transport limitations during kinetic measurements .......... 26
2.9 Supplemental electro-HFN reaction kinetics data and observations .......... 27
2.9.1 Styrene order dependences as a function of activity vs concentration .......... 27
2.9.2 Kinetic data within single-phase electrolytes .......... 27
2.10 Supplemental reaction kinetics data for side reactions .......... 28
2.11 On the conductivity of $CeO_2$ and microscopic electronic picture Rh@$CeO_2$ sites .......... 31
2.11.1 Conductivity of $CeO_2$ and electrochemical accessibility of Rh .......... 31
2.11.2 Possible effects of reactants on *operando* XANES response .......... 32
2.12 On possible reaction mechanisms .......... 33
2.13 Overpotentials and energy efficiency of electro-HFN .......... 34
3. References .......... 36

# 1. Materials and Methods

## 1.1 Materials List

All chemicals and materials were used as received unless otherwise specified.

*Catalyst materials*

- Cerium(III) nitrate hexahydrate (Beantown Chemical, 128635)
- Rhodium (III) acetylacetylonate (97%, Sigma Aldrich 282774)
- Toray 120 carbon paper (Fuel Cell store, 5% wetness proofing)
- Nafion 117 solution (5% in alcohols and water, Sigma Aldrich 70160)
- Carbon black (Vulcan XC 72, Fuel Cell Store)
- Rhodium(III) oxide nanoparticles (99.8%, Sigma Aldrich 204226)

*Reactivity Studies*

- Isopropanol (>99.5% Sigma Aldrich 190764)
- Tetrabutylammonium trifluoromethanesulfonate (Sigma Aldrich 86888)
- Trifluoromethanesulfonic acid (Sigma Aldrich 347817)
- Styrene (Sigma Aldrich, S4972)
- Carbon monoxide gas (Airgas CM R300)
- Hydrogen gas (Airgas HY UHP35)
- 1,3,5 trimethoxybenzene (>99%, Sigma Aldrich 138827)
- Neosepta AHA membrane (Ameridia Innovative Solutions)
- Aluminum foil (Reynold's Wrap)

## 1.2 Catalyst and Electrode Preparation

### 1.2.1 $CeO_2$ nanoparticle synthesis

$CeO_2$ nanoparticle synthesis was adapted from the literature.[1] In summary, 2 g of cerium(III) nitrate hexahydrate (Beantown Chemical, 128635) was ground with mortar and pestle, put in a combustion boat, and calcined under nitrogen flow at 350ºC for 2 hrs in a tube furnace. The resulting light yellow solid was ground again with mortar and pestle and stored for later use.

### 1.2.2 Rh@$CeO_2$ synthesis

Rh@$CeO_2$ was synthesized via a wet impregnation procedure that was adapted from the literature.[1] In summary, 1 g of $CeO_2$ powder was combined with 99 mg of rhodium (III) acetylacetylonate (97%, Sigma Aldrich 282774) and approx. 3 mL of acetone in a heated (~80ºC) mortar. The rhodium salt dissolved completely in acetone, and the mixture was ground with a pestle until all of the acetone evaporated. The

resulting powder was transferred to a combustion boat, dried at 80ºC for 30 minutes, and transferred to a muffle furnace, where it was heated in static air at 800ºC for 10 hours with a 10ºC/min ramp rate.

### 1.2.3 Carbon paper support preparation

Toray 120 carbon paper (Fuel Cell store, 5% wetness proofing) was punched into 13 mm diameter circles and calcined in a muffle furnace in static air at 600ºC for 1 hour. The muffle furnace treatment partially oxidizes the carbon paper surface to make it more hydrophilic.

### 1.2.4 Working electrode (Rh@$CeO_2$ on carbon paper) preparation

A catalyst dispersion solution containing solvent, Nafion (ionically conductive binder), and carbon black (conductive additive) was prepared by mixing the following: 13.2 mL isopropanol, 6 mL of Nafion 117 solution (5% in alcohols and water, Sigma Aldrich 70160), and 64 mg of carbon black (Vulcan XC 72, Fuel Cell Store).

Catalyst ink solutions were prepared by mixing Rh@$CeO_2$ powders with the above catalyst dispersion solution in a ratio of 60 mg Rh@$CeO_2$ to 960 µL of catalyst dispersion solution. The resulting catalyst ink solution was shaken and sonicated for ~5 minutes until it visually appeared well-dispersed and was then drop-casted onto hydrophilic carbon paper supports at a loading of 5 mg Rh@$CeO_2$ per carbon paper (for a loading of 3.77 mg Rh@$CeO_2$ per $cm^2$ electrode). To achieve this, approximately 35 µL of the catalyst ink solution was drop casted on each side of the carbon paper support. Electrodes were dried at 80ºC for 30 minutes and then annealed at 150ºC for 6 hours. The final annealing step was roughly based off of Nafion membrane annealing literature.[2]

## 1.3 Catalyst Characterizations

### 1.3.1 Quantifying Rh loading for Rh@$CeO_2$ particles via ICP-MS

Actual Rh loading in the Rh@$CeO_2$ catalysts was determined following a previously reported procedure involving acid digestion followed by inductively coupled plasma mass spectrometry (ICP-MS).[3] In short, around 5 mg of the powder catalyst was mixed with 5 mL of aqua regia (1 mL of concentrated $HNO_3$ added to 4 mL of concentrated HCl). The sample was then digested in a sealed, Teflon-lined Parr digestion vessel at 80ºC for more than 48 hours. The sample was then diluted into a 2% nitric acid solution, and terbium was added as an internal standard. Finally, the sample was filtered with a 0.22 µm polypropylene syringe filter (ThermoFisher Scientific, CH2213-PP), and the Rh concentration was quantified using an ICP-MS (Agilent 7900 ICP-MS).

### 1.3.2 Powder X-ray diffraction measurements

pXRD data were collected on a Rigaku SmartLab diffractometer (Cu Kα). All diffraction patterns were collected from 5-80º 2θ at a step size of 0.01º and 0.9º per minute. Data were refined using GSAS-II software.

### 1.3.3 XPS measurements

XPS data were collected using a Kratos AXIS Ultra spectrometer. Samples of Rh@$CeO_2$ and $CeO_2$ were prepared by depositing a thin layer of powder onto carbon tape. Samples were irradiated with a monochromatic Al Kα x-ray source (1486.6 eV) at 150 W at pressures $< 1 \times 10^{-9}$ Torr. The analyzer pass energy was set to 80 eV for survey scans and 10 eV for high-resolution scans. All spectra were calibrated to adventitious C at 284.8 eV. All sample measurements were taken with the neutralizer on to minimize charge buildup on insulating samples. XPS data were analyzed using CasaXPS software. During data processing in CasaXPS, all peaks used a line shape LA(50). Binding energies, full-width half-maximum values, and areas are listed for each component in the Ce 3d and O 1s spectra for Rh@$CeO_2$ and $CeO_2$ samples. The area of Ce $3d_{5/2}$ peaks were constrained by their Ce $3d_{3/2}$ counterpart, e.g. u'''/v''', in order with a 2:3 area ratio of $3d_{3/2}$ to $3d_{5/2}$. Additionally, Ce 3d peak positions were constrained by a spin–orbit splitting value of $\Delta E$(Ce 3d) = 18.6 eV.

In order to determine the stoichiometry of surface $CeO_x$ in Rh@$CeO_2$, the $Ce^{3+}/(Ce^{3+} + Ce^{4+})$ ratios were calculated by using the sum of the $Ce^{3+}$ peak areas divided by the sum of all Ce 3d peaks. These calculations predict that the surface consists of 24% $CeO_{1.5}$ and 76% $CeO_2$ with an average formula of Rh@$CeO_{1.87}$.

### 1.3.4 Synchrotron XAS measurements (*ex situ* and *operando*)

Both *operando* and *ex situ* XAS measurements were performed at the Inner Shell Spectroscopy (ISS, also known as 8-ID) beamline[4] at Brookhaven National Laboratory. ISS is a damping wiggler beamline with an energy range of 4.9 – 33 keV and a flux of $5 \times 10^{13}$ photons/s at 12 keV. Full specifics about ISS are detailed elsewhere.[4] Catalyst samples were prepared identically to as they were for actual reactivity studies. Additionally, measurements were taken using a standard electrochemical cell with the only differences being (1) for convenience, no separator was used and (2) the back plate touching the working electrode had a Kapton (X-ray transparent) window in it, and electrical contact to the working electrode was made with a thin ring of aluminum tape (i.e., aluminum tape with a 12 mm diameter hole punched in it) rather than the glassy carbon plate that was used for actual electrolysis experiments (Figure S1)

All XAS measurements on catalyst samples were collected in fluorescence mode, with the sample facing the incident beam at a 45-degree angle. Fluorescing photons were collected with a PIPS detector, and better signal-to-noise (s/n) was achieved with two strategies: (1) use of a z-1 (Ru) filter to absorb adventitious, lower-energy fluorescence and (2) use of soller slits to help focus fluorescence collection to a more localized spatial region right at the electrode surface. We did not find the use of a silicon drift detector (SDD) to help improve s/n in this case. One scan over the energy range of 23020 eV to 24212 eV (approx. 5 eV increments) was collected over the course of 35 seconds.

For *ex situ* measurements, samples were mounted inside the aforementioned XAS cell, but the cell was kept empty (i.e., no electrolyte and open to ambient air).

For any given *operando* experiment, the sample was first filled with a typical electro-HFN electrolyte: 0.1 M TBAOTf + 25 mM HOTf in either 50% or 80% v/v IPA/$H_2O$ electrolyte (we found the exact water content in the electrolyte did not affect the measurements, Figure S11c). Then, the appropriate reactants were added (between 0.4 – 0.5 M styrene when styrene was added, and 10 sccm of CO at 1 bar when CO was added). The electrode was then sequentially stepped from the least reductive to the most reductive potential. At each potential, the sample was allowed to reach steady state for 10 minutes, and then XAS data at the Rh K-edge was collected for ~30 minutes, yielding approximately 30 scans that would later be

averaged during data analysis. Each scan took approx. 1 minute, and all 30 scans were collected at different locations on the electrode, where different locations were auto-defined by a roughly square-shaped grid pattern with 1 mm spacing between locations. The beam spot size was 1 mm x 1 mm (FWHM). We collected in different locations simply to get a more spatially averaged measurement of the electrode; we tested for but did not see any evidence of beam damage (i.e., no visible changes in XAS spectra even if all 30 scans were collected at the exact same location). For the same electrolyte/gas settings, the electrode was not swapped out between different tested potentials. For different electrolyte/gas settings, the cell was disassembled, cleaned, and prepared with a fresh electrode and electrolyte. All experiments were performed at ambient pressure with gas (either CO or $N_2$) bubbling through the electrolyte.

Reference materials employed for these studies were $Rh_2O_3$ powder (Sigma Aldrich 204226) and Rh(0) foil. The $Rh_2O_3$ powder was diluted into PEG powder using quantities calculated in Athena[5] and then pressed into a pellet. XAS measurements were then collected on this pellet in transmission mode. Spectra for the Rh(0) foil were obtained by using reference channel data for an arbitrary run. Since Rh(0) foil was used as the reference channel, using $I_R$ (reference current)/$I_T$ (transmission current) gave the XAS absorption of the Rh(0) foil.

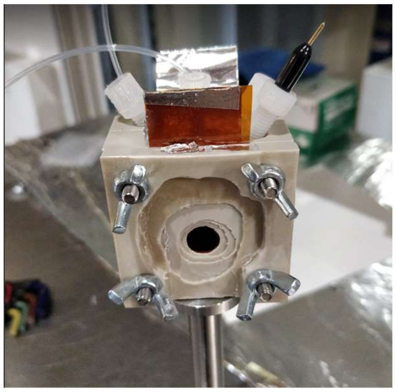

**Figure S1.** *Operando* XAS cell. Depicted is the Kapton window, which is pressed right against the back of the working electrode in an electrochemical cell that is otherwise configured identically to a typical electrolysis cell.

### 1.3.5 XANES and EXAFS data analysis

General XAS data processing was performed in Athena, and EXAFS fitting was performed using Artemis.[5]

For XANES processing, all spectra were normalized with the same parameters. Rh K-edge energies that are reported represent the energies at which the normalized spectra obtain a value of 0.5; this was intended as an approximation of the inflection point of the rising edge.

For EXAFS fitting, IFEFFIT calculations of $Rh_2O_3$ scattering paths were performed using a trigonal $Rh_2O_3$ cif structure (mp543734) downloaded from materialsproject.org. For this structure, the IFEFFIT calculations yielded two nearly identical Rh-O scattering paths, each with a coordination number of 3. For the fitting in this work, to reduce the number of fitting parameters, only the Rh-O scattering path with the shorter characteristic length was used for fitting, and for bulk $Rh_2O_3$, its associated coordination number

was set to 6. In terms of other data processing parameters, we used an rbkg value of 1.2 and constrained the fitting between r values of 1.25 and 2.15 (to get approx. the first coordination shell).

## 1.4 Reactivity Studies

### 1.4.1 Electrolyte preparation

The default electrolyte mixture used in these studies was a 50/50 v/v water/isopropanol mixture with 0.1 M tetrabutylammonium trifluoromethanesulfonate (TBAOTf, Sigma Aldrich 86888) and 0.025 M trifluoromethanesulfonic acid (HOTf, Sigma Aldrich 347817). Directly prior to performing a reaction, this electrolyte was combined with styrene (Sigma Aldrich, S4972) to afford 0.52 M styrene in the electrolyte solution. Notably, 0.52 M styrene is above the solubility limit, so the final electrolyte was cloudy with a small amount of phase separation. During order dependence studies, the styrene and HOTf concentrations were changed accordingly. Additionally, Tafel and proton order dependence studies were performed both above (0.52 M styrene) and below (0.28 M) the solubility limit of styrene.

During kinetic isotope effect studies, water, isopropanol, and triflic acid were substituted with deuterium oxide (Sigma Aldrich, 151882), isopropanol-$d_8$ (Sigma Aldrich, 175897), and DOTf (Sigma Aldrich, 369632), respectively. For experiments with 1-hexene and 1-decene, concentrations of the substrates were 1 M and 0.32 M, respectively, where concentrations were chosen to be slightly above the solubility limit of each olefin, such that a small amount of phase separation was observed in both the starting and post-electrolysis electrolytes.

### 1.4.2 Thermochemical reaction setup

Thermochemical HFN reactions were performed in 350 mL Parr reactors made of Alloy C276. All thermochemical measurements were intentionally performed such that parameters such as catalyst loading/preparation, electrolyte, reactant concentration, etc., were directly comparable to electrochemical reactions.

For ambient temperature tests, one catalyst-covered carbon paper electrode was added to a 25 mL round bottom flask, in addition to 1 magnetic stir bar and 2 mL of electrolyte solution. This round bottom flask was placed within the Parr reactor, and the Parr was then sealed. Then, the Parr reactor was purged with nitrogen gas. Afterwards, 3 pump-purge cycles were performed with CO in which the reactor was charged to 5 bar CO (Airgas CM R300), then purged, and this process was repeated for a total of 3 times. Then on the fourth pump step, the reactor was left at 5 bar CO. Subsequently, the reactor was then connected to an $H_2$ (Airgas HY UHP35) line and filled to the appropriate pressure of $H_2$ (typically 5 – 10 bar $H_2$ for a total pressure between 10 – 15 bar). The reactor was then left on a magnetic stir plate, typically overnight, to allow the reaction to occur (typically 14 – 16 hrs). Upon completion of the reaction, the gas was released, reactor purged with $N_2$, and reaction mixture within the flask was worked up (vide infra).

Reactions at elevated temperature were performed on 10 – 15 mL volume scales and directly within the body of the Parr reactor (no round bottom flask used) for more reliable temperature control. Aluminum foil, heat tape, another layer of aluminum foil, and insulation were wrapped around the exterior of the reactor in that order. The thermocouple used was directly in contact with the reaction solution inside the reactor.

### 1.4.3 Electrochemical reaction setup

*Cell and electrode configurations*

Electrochemical measurements were performed in PEEK sandwich cells (Custom-made, Lab Machinist Solutions) in a 2-compartment configuration. Aluminum foil (Reynold's Wrap) was used as the sacrificial counter electrode, Neosepta AHA membranes (Ameridia Innovative Solutions, stored in milliQ water and rinsed with IPA before use) were used as the separator, and when applicable, a leak-free Ag/AgCl electrode (Innovative Instruments LF-2) was used as the reference electrode. Anode and cathode compartments were filled with 2 mL of electrolyte solution each. A miniature magnetic stir bar was also added to the cathode compartment. Seals were made with chemically resistant Viton O-rings (McMaster-Carr 1170N32/1170N26). All structural components for the 2-comparment cells are shown in a blown-out view in Figure S2a. They were assembled as shown in the steps illustrated by Figure S2b. Between experiments, all parts were rinsed thoroughly with acetone and a new catalyst electrode and membrane were used for each condition tested.

For studies with the proton balanced cell, a Pt foil (Alfa Aesar 00262) was used as the counter electrode, and an activated Nafion membrane (Fuel Cell Earth N11730) that was pre-soaked in a water/IPA mixture was used as the separator.

*Gas delivery configurations*

The gas configuration for electrolysis experiments at elevated pressures is shown in Figure S2C. To set up an experiment, 3 pump-purge cycles were performed with CO in which the reactor was charged to 5 bar CO (Airgas CM R300), then purged, and this process was repeated for a total of 3 times. Then on the fourth pump step, the reactor was left at 5 bar CO. Notably, both catholyte and anolyte were connected to the same static atmosphere of CO – this helped ensure no large pressure differentials across the separator, and gas-phase crosstalk between anode and cathode was assumed to not influence the electrochemical hydroformylation reaction of interest. A reference electrode was not used in elevated pressure experiments.

The gas configuration for electrolysis experiments at ambient pressure is shown in Figure S2d. In short, CO gas was bubbled into the catholyte at 10 sccm. Bubbling into the catholyte was necessary to achieve high degree of convection required to mitigate mass transport limitations and allow for collection of more reliable kinetic data.

Finally, the gas configuration for the high pressure electrolysis experiments with gas flow is shown in Figure S2e. In summary, CO was flowed through the headspace of each compartment using flow controllers, and the total pressure of the cell was controlled by using a pressure regulator at the outlet of the cell. This configuration was used for all $H_2$ quantification at elevated pressures. Additionally, this was used for the proton balanced cell, so that $O_2$ generated from OER at the anode would not accumulate in the cell and cause possible explosion hazards.

*Elevated temperature experiments*

For elevated temperature experiments, the cell was wrapped with heat tape and a thermocouple was inserted into one of the Idex ports of the cell so that it was in direct contact with the catholyte.

*Details on the application of voltage/current*

Application of potential was performed using a potentiostat (Biologic VMP-3e). Elevated pressure experiments were performed as chronopotentiostatically (CP, constant current). Ambient pressure

experiments were performed chronoamerometrically (CA, constant voltage). CA measurements were run with 90% automatic resistance compensation, where electrolyte resistance was determined using potentio-electrochemical impedence spectroscopy (PEIS) measurements. The last 10% of IR drop was not compensated. Typical electrochemical experiments, at both elevated and ambient pressures, were run for 1 – 3 hrs.

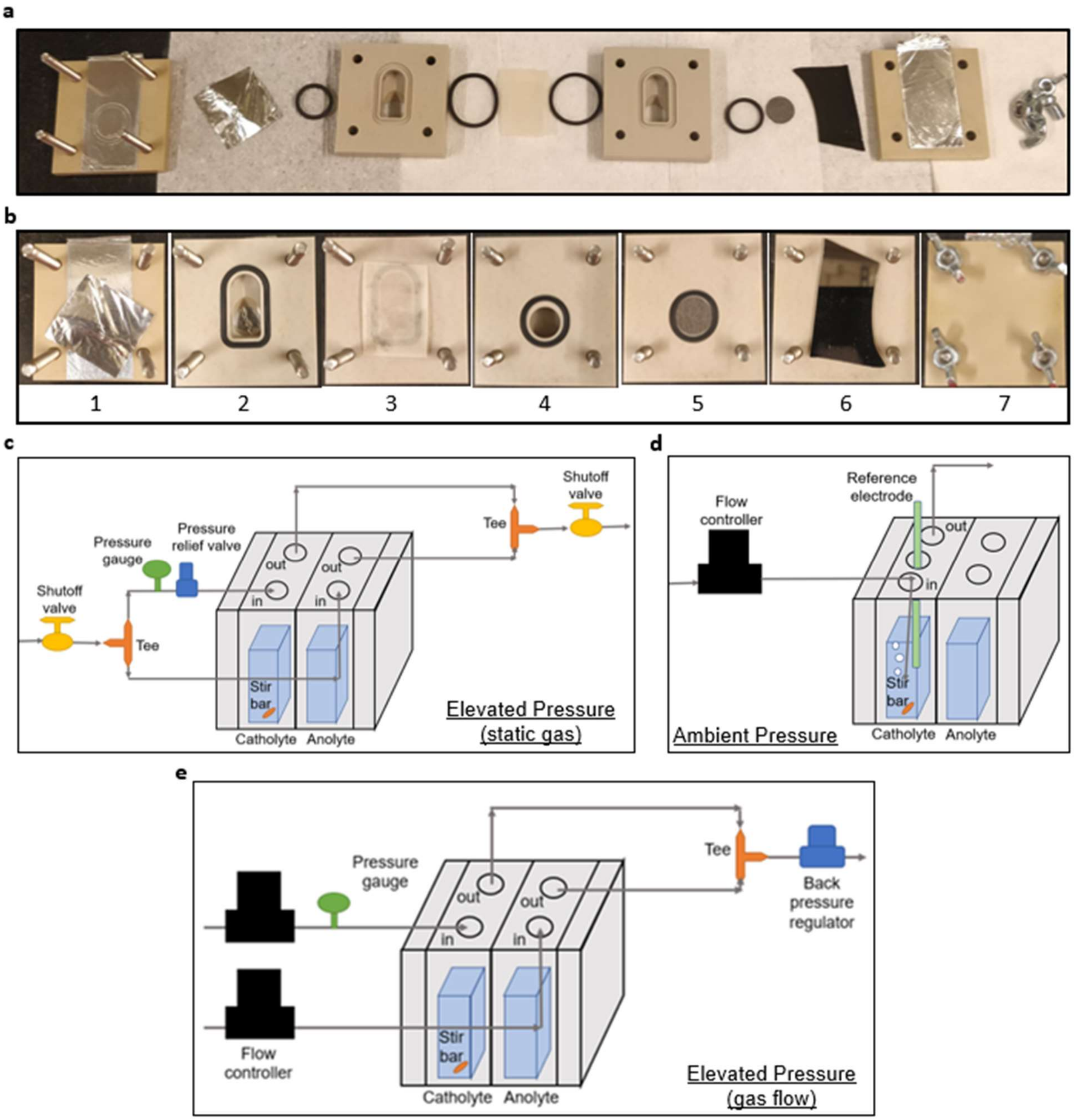


**Figure S2**. Assembly and set up of the electrochemical cell. **a**, Exploded-view picture of all cell parts. **b**, Birds-eye view of the cell as it is assembled. **c**, Gas configuration for elevated pressure experiments, in which there is no gas flow, just a static pressurized headspace. **d**, Gas configuration for ambient pressure experiments, in which gas is bubbled through the electrolyte. **e**, Gas configuration for elevated pressure experiments with gas flow through the headspace.

### 1.4.4 Reactivity studies at elevated temperatures

For thermochemical and electrochemical reactions performed at elevated temperatures, the reaction vessel (either the Parr reactor or the entire sandwich cell) was wrapped in heat tape. A thermocouple was introduced directly into the reaction solution. For the thermochemical reactions, this thermocouple was protected by a shell of Alloy C-276, and for the electrochemical reactions, the thermocouple was coated in a protective layer of Teflon. For the thermochemical reaction, the reactor was fully charged with reactants before heating started. Then, the reactor was allowed to heat for ~20 minutes to the desired setpoint, and then a timer measuring reaction time was set. For the electrochemical reaction, the reactor was given ~15 minutes to heat up to the desired setpoint before current was applied.

### 1.4.5 Reaction workup and quantification of liquid phase products

For thermochemical measurements, the entire electrolyte was collected for product analysis, and for electrochemical measurements, only the catholyte was collected for product analysis (product crossover to the anolyte was confirmed to be negligible). In both cases, this yielded approx. 2 mL of collected liquid. To the reaction liquid, 20 µL of 0.16 M 1,3,5-trimethoxybenzene (TMB, Sigma 138827) solution in IPA was added as an internal standard. Then, 1 mL of acetonitrile and 1 mL of milliQ water were added. Finally, the reaction was extracted three times, using 500 µL of hexanes for each extraction. A Gas Chromatograph-Mass Spectrometer (GCMS, 7890B GC, Agilent) fitted with a DB-WAX column and Flame Ionization Detector (FID) was used to identify and quantify products. Splitless injection was used. The temperature ramp of the GCMS method is shown below. GCMS analysis was performed directly on the ~1.5 mL of collected organic phase.

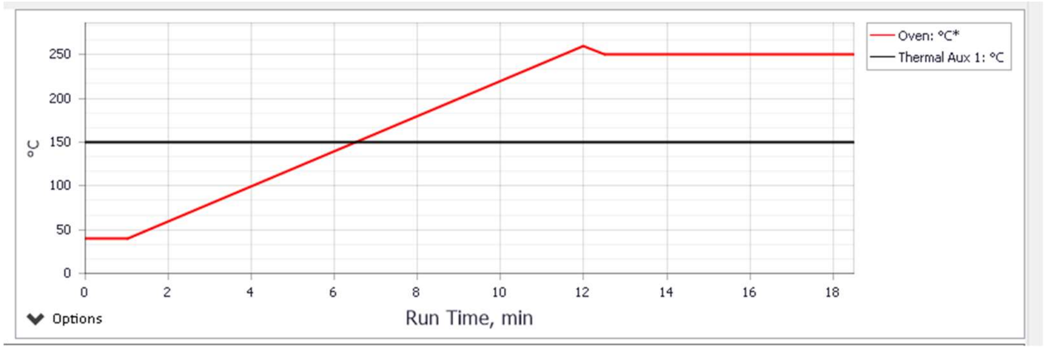


**Figure S3.** GC temperature ramp method.

For quantitative analysis of the FID signal, we constructed calibration curves to account for two unknowns: (1) the FID response factor differences between the internal standard (TMB) and the products and (2) differences in extraction efficiencies between the internal standard and the products (we noticed some product remained in the aqueous phase during extractions). To account for both of these factors simultaneously, we constructed a calibration curve in which we added known amounts of the products (2-phenylpropanal (Sigma 241369) and/or ethylbenzene (Sigma 296848)) to 2 mL of electrolyte solution and worked up the resulting "product-spiked electrolyte" solution in the same way that we worked up electrolyte collected from actual reactions (as detailed in the paragraph above). Integrations from the resulting FID spectra yielded calibration curves with "observed product/TMB peak ratio" on one axis and "known product/TMB molar ratio prior to extraction" on the other. The number of moles in the calibration curve for 2-phenylpropanal ranged from around 60 – 800 µmoles in 2 mL of electrolyte, and the calibration curve

for ethylbenzene ranged from 0.5 – 1.2 mmoles in 2 mL of electrolyte (the quantification for ethylbenzene was less sensitive than that for 2-phenylpropanal). Finally, to account for any errors in massing during the preparation of reference solutions with known product amounts, a second set of solutions was prepared identically to the "product-spiked electrolyte", but with 2 mL of $CDCl_3$ instead of electrolyte. This resulting solution was directly analyzed with NMR to confirm molar ratios between products and internal standard.

### 1.4.6 Quantification of hydrogen gas evolution

Hydrogen gas was quantified using an online gas chromatograph (GC, 8610C SRI MultiGas 5). Samples were injected through a 1 ml sample loop to a mol sieve column held at 80°C. A thermal conductivity detector (TCD) was used to quantify hydrogen. The sample sequence was run every 5 minutes. Hydrogen FE was calculated by averaging $H_2$ signal from the 5 minute mark until the end of the run.

### 1.4.7 Calculation of turnover frequency and faradaic efficiency

Turnover frequency was calculated as following:

$$(moles\ of\ product) \Big/ (moles\ of\ active\ sites) \cdot (reaction\ time)$$

The products of electro-HFN were 2-phenylpropanal, and, at elevated temperatures, 3-phenylpropanal as well (the latter was not detected at room temperature). The number of active sites was calculated assuming all deposited Rh sites were active. This may be an overestimation of the actual number of catalytically accessible Rh atoms, so the TOFs reported in this work may be an underestimation of the true TOF of the system. Because this site normalization was used consistently throughout the work, its nature does not change any mechanistic interpretations of the data.

Faradaic efficiency was calculated as the following:

$$F * \ (moles\ of\ product) \cdot (\#electrons\ per\ product) \Big/ (total\ charge\ passed)$$

Where F is Faraday's constant, and all products reported in this work (electro-HFN, hydrogenation, and HER) are 2 electron products.

### 1.4.8 Experimental measurements of styrene and proton activity

For styrene order dependence studies, the activity of styrene was experimentally measured using methods previously reported for quantification of water activity in mixed electrolytes.[6] In short, 4 mL of representative styrene-containing electrolyte samples were added to 20 mL headspace sampling vials under ambient conditions and then capped. The vials were then vigorously shaken and allowed to equilibrate for at least 8 hours. 4 mL of pure styrene was used as the activity equals 1, pure liquid phase reference. Headspace GC-TCD was then used to quantify the vapor pressure of styrene in the various samples. The activity of styrene for any given sample was simply calculated as:

Activity = (styrene peak area of sample)/(styrene peak area of pure styrene sample)

While previously reported methods were used for water activity quantification and yielded large variability for the tested organic substrate (cyclohexanone, bp = 155.6°C), we found this procedure to yield reproducible results for styrene (bp = 145°C), possibly due to styrene's lower boiling point.

A plot of styrene concentration vs measured activity is shown in Figure S4a. The points at 0.39 M and 0.54 M correspond to systems above the solubility limit of styrene, where phase separation has occurred. The reason why the activity curve does not reach 1 at the point of phase separation is because in a phase-separated system, while the styrene-rich phase is enriched in styrene, some amount of salt, acid, and isopropanol are expected to partition into this phase. Thus it has a styrene chemical potential lower than that of the pure styrene reference.

To quantify proton activity, reversible hydrogen electrode (RHE) potentials for electrolytes with different HOTf concentrations were also measured using methods based on literature.[7–9] In short, RHE was determined by measuring steady-state open circuit voltage (OCV) a Pt/C carbon electrode with 1 atm of $H_2$ gas bubbling. Measured RHE potentials are reported in Figure S4b.

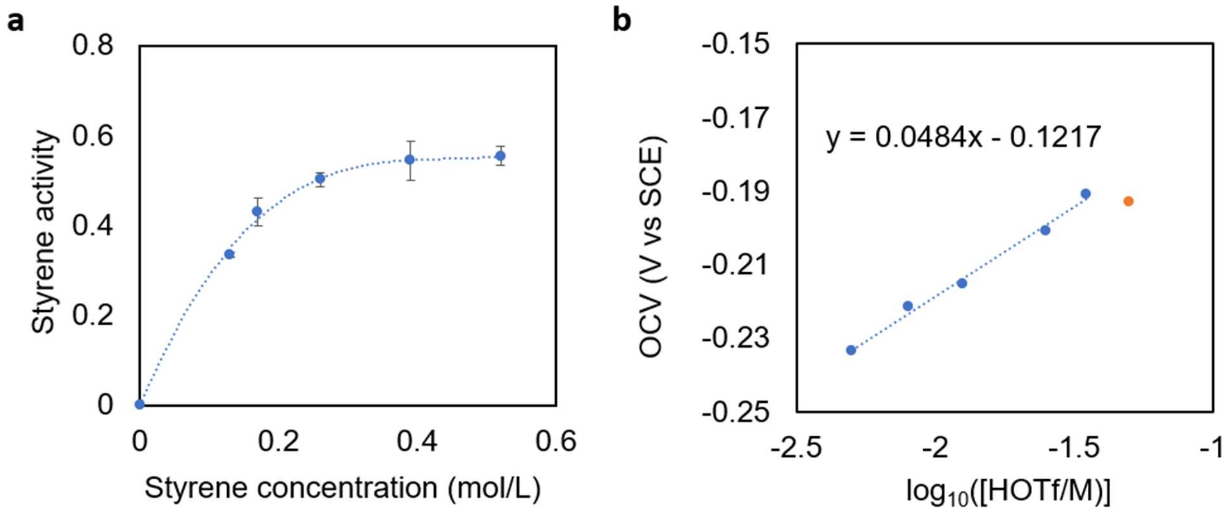


**Figure S4.** Characterization of liquid-phase species activity in electrolytes used for order dependence studies. **a**, Experimentally measured styrene activity as a function of styrene concentration. All points were collected in triplicate and error bars indicate population standard deviation. **b**, experimentally measured reversible hydrogen electrode (RHE) potentials under 1 bar of $H_2$ gas flow for electrolyte with different concentrations of HOTf. In all cases, electrolyte consisted of 50% v/v $H_2O$/IPA with 0.1 M TBAOTf, 0.52 M styrene, and 25mM HOTf (unless explicitly labeled otherwise).

# 2. Supplemental data and discussion

## 2.1 Supplemental catalyst characterization

### 2.1.1 Transmission electron microscopy

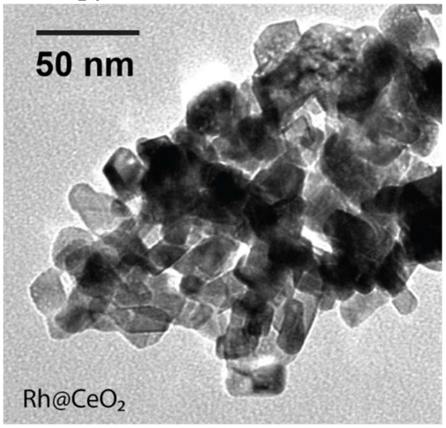


**Figure S5**. TEM image of as-synthesized Rh-impregnated $CeO_2$ (Rh@$CeO_2$) particles. Individual $CeO_2$ nanoparticle sizes appear to be ~20 nm.

### 2.1.2 X-ray photoelectron spectroscopy

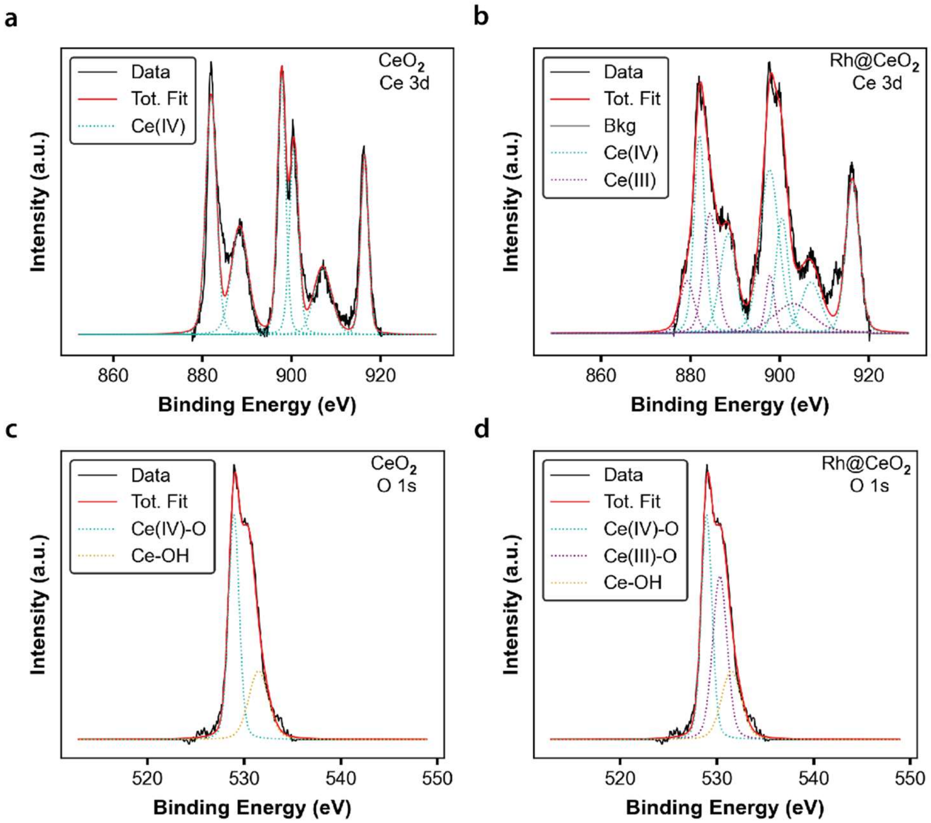


**Figure S6**. XPS spectra. Shown are the Ce 3d spectra of **a**, $CeO_2$ and **b**, Rh@$CeO_2$, as well as the O 1s spectra of **c**, $CeO_2$ and **d**, Rh@$CeO_2$. Peak deconvolutions are shown in dotted cyan (for Ce(IV)$O_2$-derived contributions) and magenta (for Ce(III)$_2O_3$-derived contributions). Total fits are shown in solid red. Analysis of the relative contributions from Ce(III) and Ce(IV) from panel **b** gives 25.8% Ce(III) defects within the Rh@$CeO_2$ material.

| Component | Peak Assignment | Binding Energy (eV) | FWHM | Area |
|---|---|---|---|---|
| $Ce^{4+}{}_{3/2}$ | u''' | 916.4 | 3.6 | 4911.1 |
| $Ce^{4+}{}_{3/2}$ | u'' | 907.1 | 5.2 | 2356.3 |
| $Ce^{3+}{}_{3/2}$ | u' | 903.1 | 10.0 | 2576.0 |
| $Ce^{4+}{}_{3/2}$ | u | 900.6 | 3.3 | 3425.5 |
| $Ce^{3+}{}_{3/2}$ | $u^0$ | 897.9 | 2.3 | 1175.2 |
| $Ce^{4+}{}_{5/2}$ | v''' | 897.8 | 5.0 | 7366.7 |
| $Ce^{4+}{}_{5/2}$ | v'' | 888.5 | 4.3 | 3834.8 |
| $Ce^{3+}{}_{5/2}$ | v' | 884.5 | 3.6 | 3864.0 |
| $Ce^{4+}{}_{5/2}$ | v | 882.1 | 2.9 | 5138.2 |
| $Ce^{3+}{}_{5/2}$ | $v^0$ | 879.3 | 3.8 | 1762.9 |
| | | | | |
| $Ce^{4+}$% | | | | 74.2% |
| x in $CeO_x$ | | | | 1.87 |

**Table S1.** XPS peak data for Ce 3d spectra of Rh@$CeO_2$.

| Component | Binding Energy (eV) | FWHM | Area |
|---|---|---|---|
| $Ce^{4+}$–O | 529.0 | 1.31 | 2138.0 |
| $Ce^{3+}$–O | 530.3 | 1.76 | 2088.5 |
| Ce–OH | 531.5 | 2.41 | 1193.8 |

**Table S2.** XPS peak data for O 1s spectra of Rh@$CeO_2$.

| Component | Peak Assignment | Binding Energy (eV) | FWHM | Area |
|---|---|---|---|---|
| $Ce^{4+}{}_{3/2}$ | u''' | 916.4 | 2.1 | 4583.8 |
| $Ce^{4+}{}_{3/2}$ | u'' | 907.0 | 4.7 | 3877.9 |
| $Ce^{4+}{}_{3/2}$ | u | 900.6 | 2.4 | 5572.9 |
| $Ce^{4+}{}_{5/2}$ | v''' | 897.8 | 2.2 | 6785.7 |
| $Ce^{4+}{}_{5/2}$ | v'' | 888.4 | 4.4 | 5816.9 |
| $Ce^{4+}{}_{5/2}$ | v | 882.0 | 2.8 | 8359.3 |

**Table S3.** XPS peak data for Ce 3d spectra of $CeO_2$.

| Component | Binding Energy (eV) | FWHM | Area |
|---|---|---|---|
| $Ce^{4+}$–O | 529.2 | 1.3 | 3783.1 |
| Ce–OH | 531.1 | 3.4 | 2390.5 |

**Table S4.** XPS peak data for O 1s spectra of $CeO_2$.

### 2.1.3 Powder X-ray diffraction

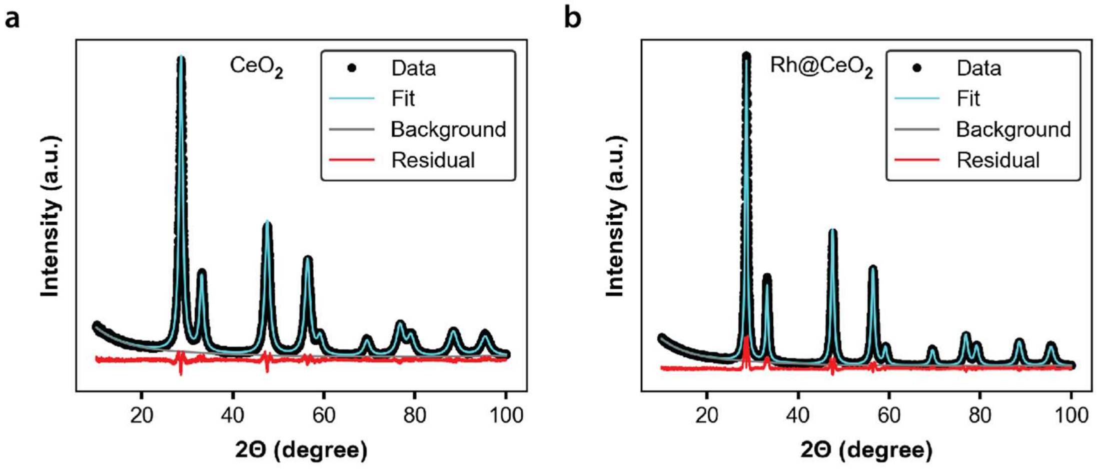


**Figure S7.** pXRD spectra of **a**, $CeO_2$ particles as-synthesized, and **b**, of $Rh@CeO_2$ catalyst. Similarity of the two spectra suggest that Rh is well-dispersed within the $Rh@CeO_2$ material.

## 2.2 Characterizations during sequential electrification and reaction optimization

### 2.2.1 Screening heterogenized Rh catalysts

When screening the heterogeneous Rh-at-metal oxide motif, we prepared several catalyst formulations using commercially available $\gamma$-$Al_2O_3$ (Inframat 26R-0804UPG) and $TiO_2$ (Aeroxide P25, Acros Organics 384290500) nanoparticles. These catalysts were prepared using methods identical to those used for $Rh@CeO_2$, but with the different metal oxide nanoparticle. Across two different Rh weight loadings, we found $Rh@Al_2O_3$ and $Rh@TiO_2$ to be more active than $Rh@CeO_2$ for thermo-HFN of styrene (Figure S8). However, $Rh@Al_2O_3$ and $Rh@TiO_2$ were not active for electro-HFN. We did not systematically investigate the reason for this, though we speculate that the lack of electro-HFN activity may be in part due to the more insulating nature of $TiO_2$ and $Al_2O_3$, but may also be due to different particle sizes – we visually observed that the $Rh@CeO_2$ was the finest powder catalyst and deposited the most uniformly onto electrode surfaces.

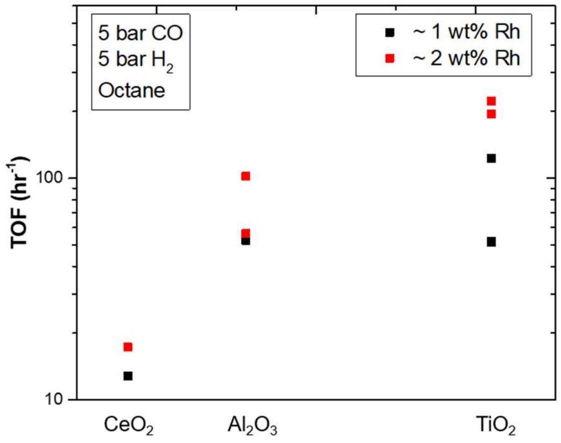


**Figure S8.** Turnover frequency (TOF) towards thermochemical hydroformylation of styrene at $CeO_2$, $Al_2O_3$, and $TiO_2$, for two different weight loadings. Rh weight percent was estimated based on mass ratio between the Rh precursor and metal oxide nanoparticle that was used during the catalyst synthesis. TOF was estimated using this estimated Rh weight percent. Thermo-HFN conditions were at 5 bar CO, 5 bar $H_2$, 80ºC, in pure octane solvent.

### 2.2.2 Finding ionically conductive media

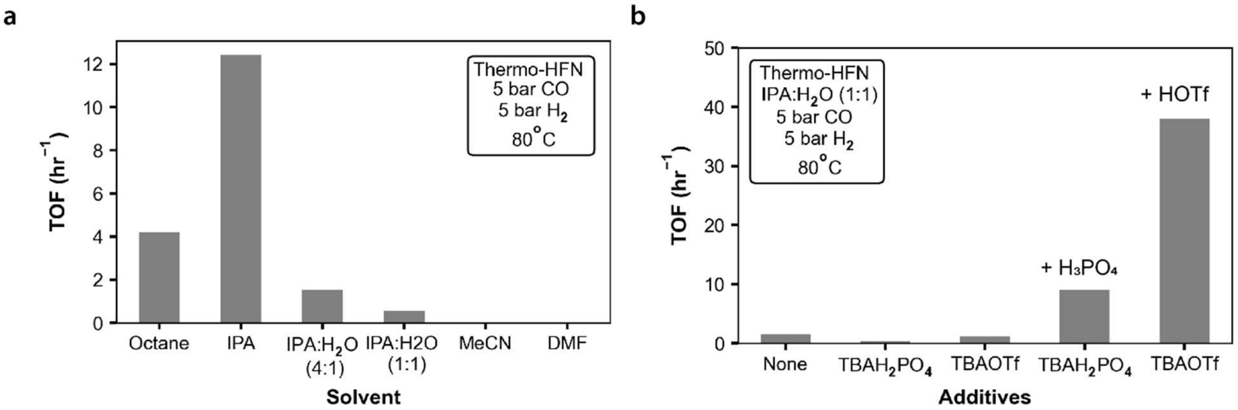


**Figure S9.** Screening for ionically conductive media conducive to thermo-HFN. **a**, solvent dependence of thermo-HFN catalyzed by powder Rh@$CeO_2$ catalysts. **b**, dependence of thermo-HFN rates on salt and acid additives in a 50% v/v IPA and $H_2O$ mixture. All rates are of styrene hydroformylation to both 2-phenylpropanal and 3-phenylpropanal. Experiments performed at 80ºC, 5 bar CO, 5 bar $H_2$, and 0.2 M styrene.

### 2.2.3 Electro- vs thermo- HFN at elevated temperatures

At elevated temperatures of 80ºC, we observed that the Rh@$CeO_2$ catalyst was fairly active for thermo-HFN, with a TOF of around 40 $hr^{-1}$ in representative electrolyte conditions, at 10 bar of 1:1 CO:$H_2$ (Figure S9c). We found activity for electro-HFN at 80ºC in comparable solvent conditions to be quite poor, with a maximum observed TOF of less than 0.1 $hr^{-1}$ (Figure S10d).

The observations at 80ºC are in contrast with what we observed at room temperature, where the thermo-HFN TOF was around 0.01 $hr^{-1}$ (Figure 1d) and the electro-HFN TOF was around 0.7 $hr^{-1}$ (Figure 1e).

The increase in thermo-HFN TOF at higher temperatures intuitively makes sense and corresponds to a positive enthalpy of reaction. The apparent decrease in electro-HFN TOF at higher temperatures likely does not hold any significant fundamental meaning. We hypothesize that the apparent decrease in rate is simply due to a greater degree of side reactivity and product degradation at elevated temperatures. From a fundamental perspective, it is likely that the electro-HFN mechanism is simply less sensitive to temperature than the thermo-HFN mechanism (lower apparent activation enthalpy), which allows for reasonable electro-HFN reactivity to be retained even at ambient temperature.

We also note changes in regioselectivity trends that occurred upon changing temperature. For thermo-HFN, we observed around 50 – 70% selectivity towards the linear aldehyde (Figure S10e) at 80ºC, but no linear aldehyde was observable at 25ºC. This observation is consistent with other observations in the field that thermos-HFN linear selectivity decreases with temperature.[10] For electro-HFN, we dominantly observe the branched product at elevated (Figure S10f) temperatures, and only observe the branched product at ambient temperatures.

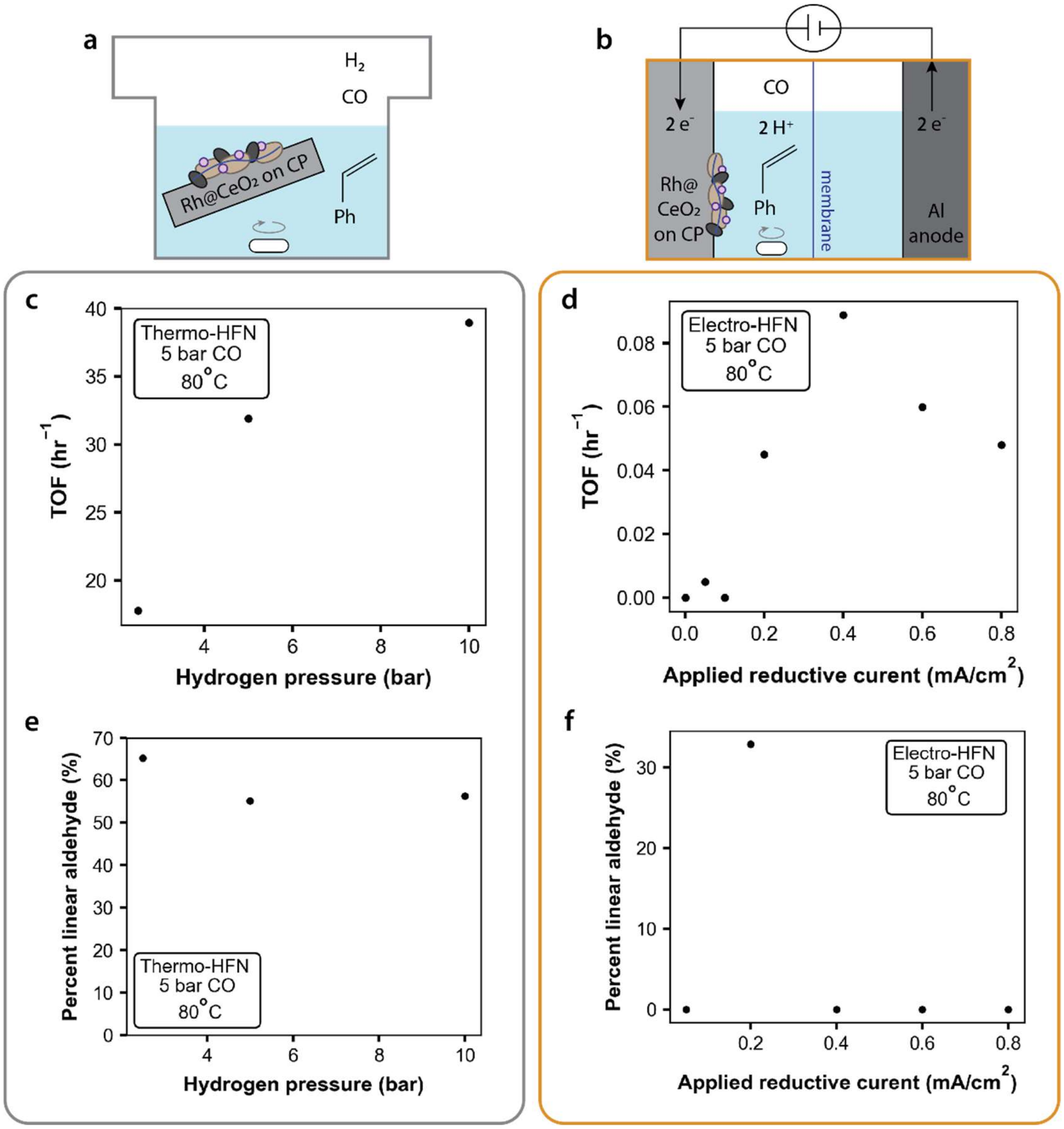


**Figure S10**. Comparison of thermo-HFN vs electro-HFN reactivity at 80ºC. Reactor configurations for **a**, thermo-HFN and **b**, electro-HFN. **c**, turnover frequency (TOF) as a function of $H_2$ partial pressure for thermo-HFN. **d**, TOF as a function of applied reductive current for electro-HFN. **e**, regioselectivity for thermo-HFN and **f**, regioselectivity for electro-HFN. Experiments performed at 80ºC, 5 bar CO, 80% v/v IPA with 20% v/v H2O, 0.1 M TBAOTf, 25 mM HOTf, and 2 M styrene.

## 2.2.4 Optimizing water content for electro-HFN at ambient temperature

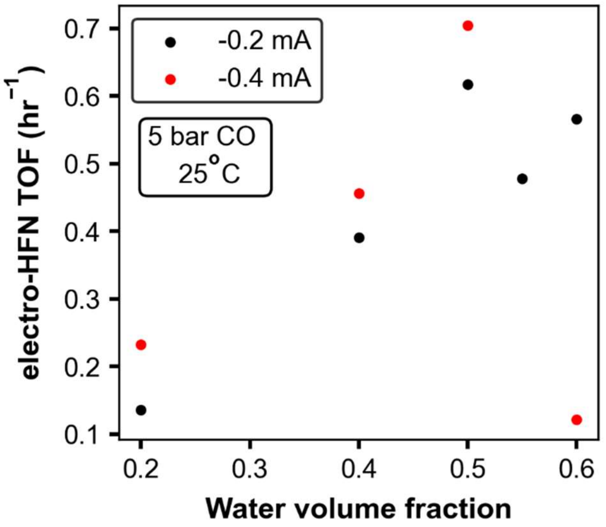


**Figure S11**. Electro-HFN rate as a function of water volume fraction for galvanostatic experiments at applied current densities of –0.2 mA/cm$^2$ (black) or –0.4 mA/cm$^2$ (red). Experiments performed at 80ºC, 5 bar CO, 0.1 M TBAOTf, 25 mM HOTf. At different water volume fractions, the styrene concentration was chosen to just exceed the saturation limit.

## 2.3 Faradaic Efficiency closure

In electro-HFN experiments, typically 20 – 40% of the Faradaic efficiency (FE) remains unaccounted for. Below we discuss the extent to which different reactions are likely to account for this missing FE.

Oxygen reduction unlikely accounts for missing FE

Adventitious oxygen could be reduced at the cathode via the oxygen reduction reaction (ORR) and lead to FE loss with only water as the byproduct. We did see some adventitious $O_2$ in our 5 bar cell during online GC measurements in our high pressure cell (flow configuration in Figure S2e). An example chromatogram showing $O_2$ detected via the thermal conductivity detector (TCD) is provided in Figure S12. Quantification of this GC signal suggests an $O_2$ concentration of around 700 ppm, which at a total cell pressure of 5 bar, corresponds to 0.0035 bar of $O_2$ partial pressure. In a previous publication, the $O_2$ transport limited current density of ORR in aqueous electrolytes at 0.5 bar $O_2$ partial pressure was estimated to be 0.26 mA/cm$^2$ (with a 200 μm boundary layer).[11] The solubility of $O_2$ in a pure aqueous solution vs a mixture of water and 1-propanol is expected to deviate by no more than 5%.[12] Thus, linearly extrapolating the above value to 0.0035 bar $O_2$ gives a transport-limited ORR current of –0.002 mA/cm$^2$, which corresponds to around or less than 1% FE for galvanostatic experiments run at around –0.2 or to –0.8 mA/cm$^2$, which is the bulk of the data presented in Figure 1f. Thus, ORR is unlikely the major sink of the missing FE.

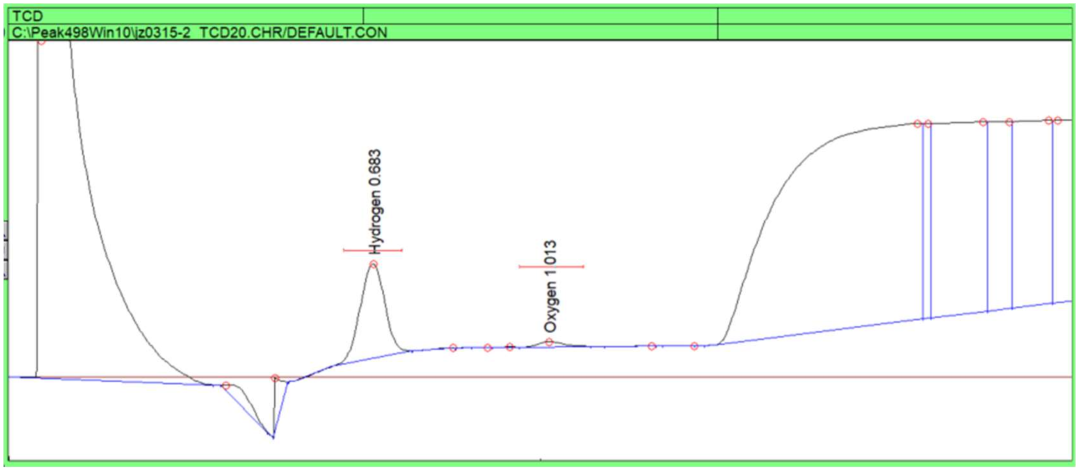


**Figure S12**. Example TCD chromatogram showing peaks for both $H_2$ and $O_2$. $H_2$ is generated via HER at the cathode, but $O_2$ is due to adventitious $O_2$ in the system.

CO reduction unlikely accounts for missing FE

CO could participate in CO reduction reactions (CORR) to make products such as methanol or ethylene. However, this is unlikely because to our knowledge, Rh-based catalysts are not considered selective for CO or $CO_2$RR.[13] Examples that use Rh also require Cu.[14] It is thus unlikely that CORR could account for >20 % FE at an Rh@$CeO_2$ catalyst.

Aldehyde product degradation unlikely accounts for missing FE

We thought that further reductive degradation of the aldehyde product might account for the missing FE. However, product stability control tests with a known amount of product dosed in under $N_2$ flow suggest that at reductive potentials, only around 10% of the aldehyde product degrades upon application of applied voltage (Figure S19). If this were via a 2-electron reductive pathway, this side reactivity would only account for up to 3% FE. While invoking a degradation pathway that uses more electrons would increase this number, it seems unlikely that aldehyde degradation accounts for a significant portion of the missing FE.

Organic side reactions of styrene may account for missing FE

Finally, we see evidence of several other side products that likely correspond to side reactions of styrene (Figure S13). Many of these peaks in the GCMS remain unidentified, but some of the identified species are shown in Figure S13. Of the identified species, the styrene dimerization product (Figure S13a) likely results from a two proton, two electron reduction. The mechanisms to arrive at benzaldehyde (Figure S13b) and acetophenone (Figure S13c) are unclear, particularly because the membrane within the cell should prevent any anodic side reactions from leading to degradation products on the cathodic side. Some amount of benzaldehyde is present in the styrene starting material, though the final amount of benzaldehyde detected varies depending on reaction conditions. The cumulative areas under these three peaks, as well as the other unidentified peaks, can correspond to about two times the area under the product peak. Assuming approximately a 1 – 2 x FID response factor (due to approx. 1 – 2 x MW) and 2 – 4 electron processes, these side products may account for up to 10 – 30% FE.

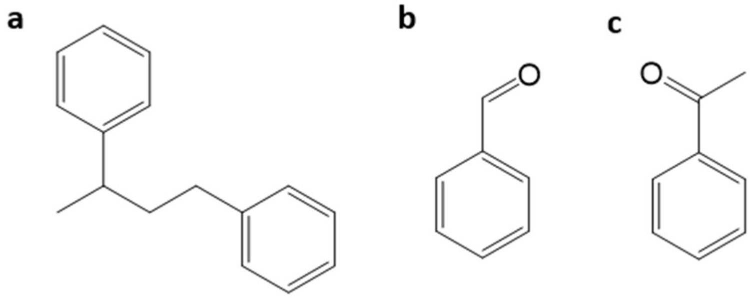


**Figure S13**. Several identified side products. While the dimerization product in **a** likely corresponds to a 2 electron, 2 proton reduction, the mechanisms to arrive at products **b** and **c** are unclear.

## 2.4 Control experiments on the nature of the electro-HFN site during catalysis

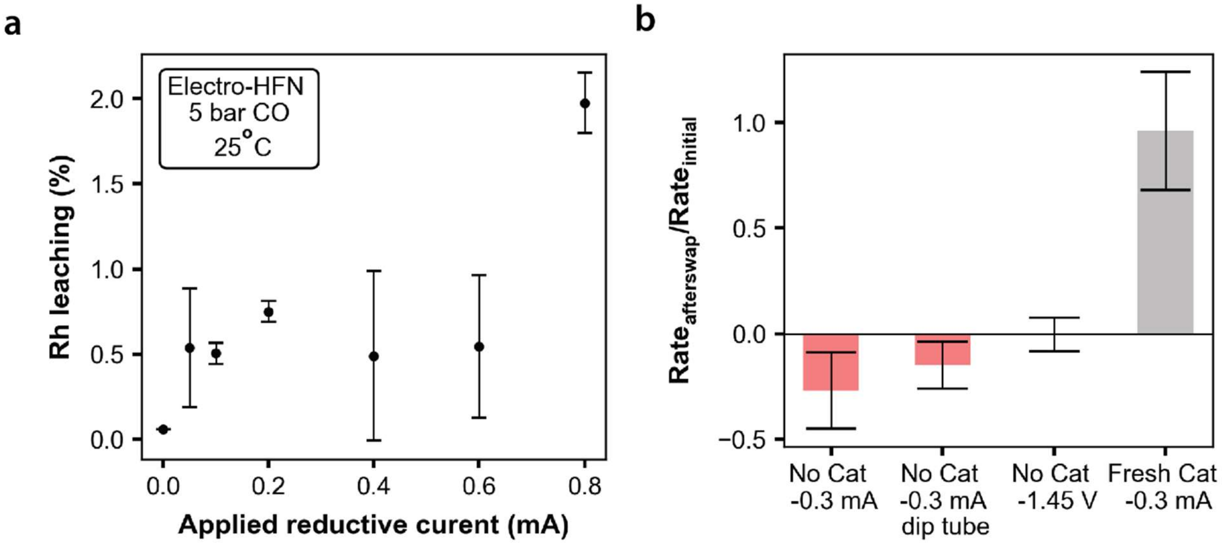


**Figure S14.** On the homogeneous vs heterogeneous of Rh sites during catalysis. **a**, Amount of solubilized Rh species detected in the electrolyte after electrolysis, quantified via ICP-MS, expressed as a percentage of the total amount of Rh initially deposited on the electrode. These data were collected at 5 bar CO, 25ºC, 2 M styrene, and 80% v/v IPA with 20% v/v $H_2O$, 0.1 M TBAOTf, 25 mM HOTf. **b**, heterogeneity test data showing relative rate after a catalyst-coated electrode was swapped with either carbon paper (denoted as "No Cat") or fresh catalyst (denoted as "Fresh Cat"). This test was done under galvanostatic conditions at –0.3 mA and 5 bar CO (bars 1, 2, and 4), or under potentiostatic conditions at –1.45 V vs SCE with 1 bar CO (bar 3). The "dip tube" label indicates that during the electrode swap, electrolyte was removed from the cell while potential was still being applied, whereas for the rest of the experiments, the potential was first stopped, then the electrolyte was removed, and then the electrode swapped. Error bars represent standard deviation with $n \geq 3$.

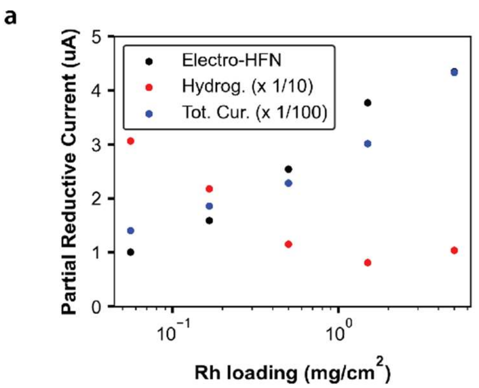


b

| Condition | Electro-HFN (µA) | Hydrogenation (µA) | Total Current (µA) |
|---|---|---|---|
| Bare carbon paper (CP) | 0 | 0 | -70 |
| CP + Nafion | 0 | 0 | -70 |
| CP + Nafion + carbon black (CB) | 0 | -50 | -150 |
| CP + Nafion + CB + $CeO_2$ | 0 | -40 | -300 |

**Figure S15.** On the catalytic contributions of each component of the working electrode. **a**, Rh content dependence of electro-HFN (black), hydrogenation (red), and total current (proxy for hydrogen evolution, or HER, blue). An increase in Rh content indicates an increase in the mass of Rh@$CeO_2$ deposited on the electrode (and not on the inherent Rh mass loading of the Rh@$CeO_2$ particles). **b**, data showing the contributions to electro-HFN, hydrogenation, and total current associated with the non-Rh-containing components of the working electrode, such as $CeO_2$, carbon black, Nafion, and carbon paper. Collected at 1 bar CO, 25ºC, –1.45 V vs SCE, 0.52 M styrene, 0.1 M TBAOTf, 25 mM HOTf, in 50% v/v IPA/$H_2O$. Data largely represents singlicate experiments.

## 2.5 Transport analysis of maximum local $H_2$ accumulation

We will analyze the case in which soluble $H_2$ is being generated at the cathode at a flux of –0.2 mA/cm$^2$, where this current density is given with respect to the planar geometric area of the electrode. The question here is whether the local concentration of $H_2$ under this current density can exceed the bulk concentration of $H_2$ expected under 10 bars of $H_2$ headspace. This analysis will help us understand whether electro-HFN rates reported in Figure 1c and thermo-HFN rates reported in Figure S9a can be consistent with an indirect electro-HFN mechanism. In the end, we will conclude that an indirect mechanism via local $H_2$ accumulation is unlikely.

We will analyze the transport problem as a constant flux boundary condition applied to a mass transport boundary layer. Since the flux across this boundary layer must be constant and equal to the boundary condition, we can write:

$$C_{bulk} - C_{interface} = \frac{nF\delta i}{D}$$

Where n is the number of electrons transferred in the reaction, F is Faraday's constant, δ is the hydrodynamic boundary layer thickness, i is the applied current, D is the diffusivity of the species, and $C_{bulk}$ and $C_{interface}$ are the bulk and interfacial concentrations of the species.

If we assume the diffusion coefficient for $H_2$ is 5E–5 $cm^2/s$ (its value in pure water at 25ºC), and that the boundary layer thickness is about 200 μm, we get:

$$C_{bulk} - C_{interface} = \frac{(2)\cdot(96485)\cdot(2E-2\ cm)\cdot(0.2\ \frac{mA}{cm^2})}{5E-5\ \frac{cm^2}{s}} = 10\ \text{mM}$$

The solubility of $H_2$ gas in pure water at 25ºC and 1 bar is 0.00016 g $H_2$/100 g $H_2O$,[15] or 0.8 mM; at 10 bar $H_2$, assuming linear scaling that is typical of gas-liquid equilibrium, the solubility of $H_2$ should be around 8 mM. For a pure alcohol such as 1-propanol at 10 atm, the solubility of $H_2$ gas is about 30 mM (extrapolated from 0.0031 mole fraction at 1.27 MPa).[16]

These solubility values are comparable to the maximum $H_2$ concentration that could be generated from a –0.2 $mA/cm^2$ reaction flux, calculated above. This suggests that an electrochemical experiment at an applied current of –0.2 $mA/cm^2$ and a thermochemical experiment with an added $H_2$ pressure of 10 bar should have approximately similar local concentrations of dissolved $H_2$, yet the rates observed for hydroformylation differ by an order of magnitude.

## 2.6 No evidence for non-Faradaic promotion

We tested for evidence that voltage could non-Faradaically promote of thermo-HFN, since this could be an alternative explanation to our kinetic data that suggested a non-mediated mechanism. It was not possible to directly test for non-Faradaic promotion of thermo-HFN at room temperature because observed electro-HFN rates exceeded those of thermo-HFN. Therefore, upon application of voltage, it would not be possible to examine only the contribution of reactivity that could be unambiguously attributed to thermo-HFN. However, at elevated temperatures, we observed that thermo-HFN was much faster than electro-HFN. Thus, at 80ºC, we could run a thermo-HFN reaction with applied potential, and rigorously attribute most or all of the observed reactivity to thermo-HFN (in these conditions, the amount of charge passed was much lower than the amount of product observed). We note that of course the mechanisms at 25 and 80ºC may not be the same.

Figure S16b shows that under thermo-HFN conditions, the measured thermo-HFN TOF does change in response to applied potential (black points). However, if the catalyst is simply dispersed in the solution, rather than being attached to the electrode (Figure S16a), the measured thermo-HFN rate responds to applied potential with a very similar trend (red points). Thus, the changes in thermo-HFN reaction rates that we observe in Figure 4b are likely not due to non-Faradaic promotion; rather, they likely arise from bulk changes in electrolyte composition that arise due to Faradaic processes at the electrode surfaces. Thus, in this experiment, we do not see compelling evidence that voltage non-Faradaically promotes thermo-HFN.

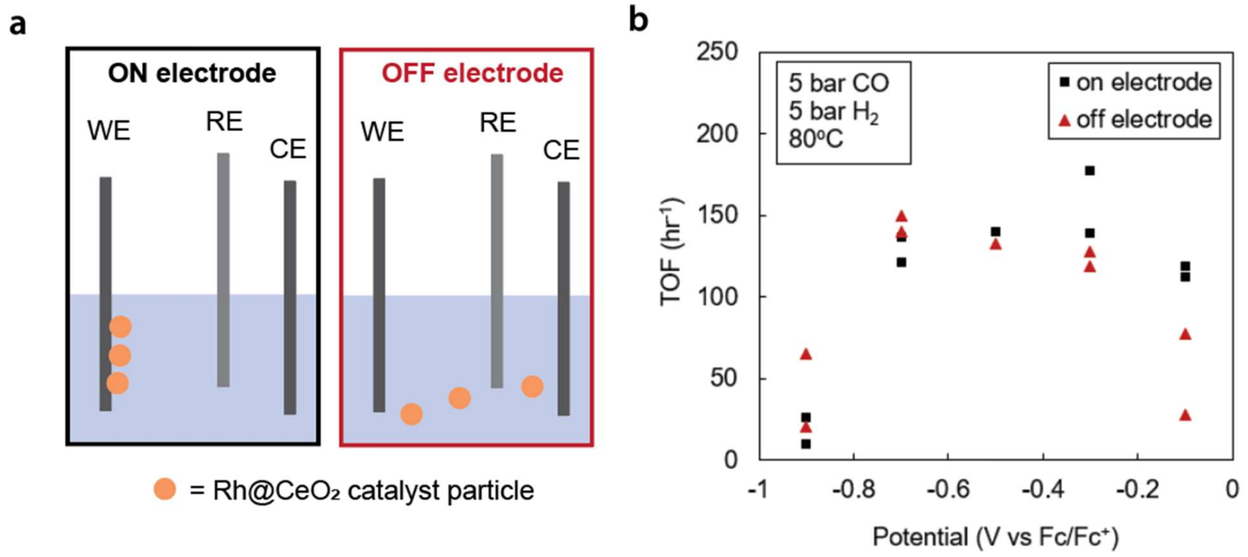


**Figure S16.** Testing for non-Faradaic promotion of electro-HFN. **a**, schematic showing the relevant reaction setups, where in one case, the Rh@$CeO_2$ catalyst particles are deposited at the working electrode (WE) (termed "on electrode"), and in the other case, the catalyst particles are free floating in the solution (termed "off electrode"). In both cases, voltage is applied at the WE, which is referenced to the reference electrode (RE). Current also passes through the counter electrode (CE), which consists of sacrificial Al foil. **b**, rate of electro-HFN as a function of applied potential for both on and off electrode configurations. Data collected at 80ºC, 5 bar $H_2$, 5 bar CO, 0.2 M styrene, 80% v/v IPA with 20% v/v $H_2O$, 0.1 M TBAOTf, 25 mM HOTf.

## 2.7 Supplemental XAS data

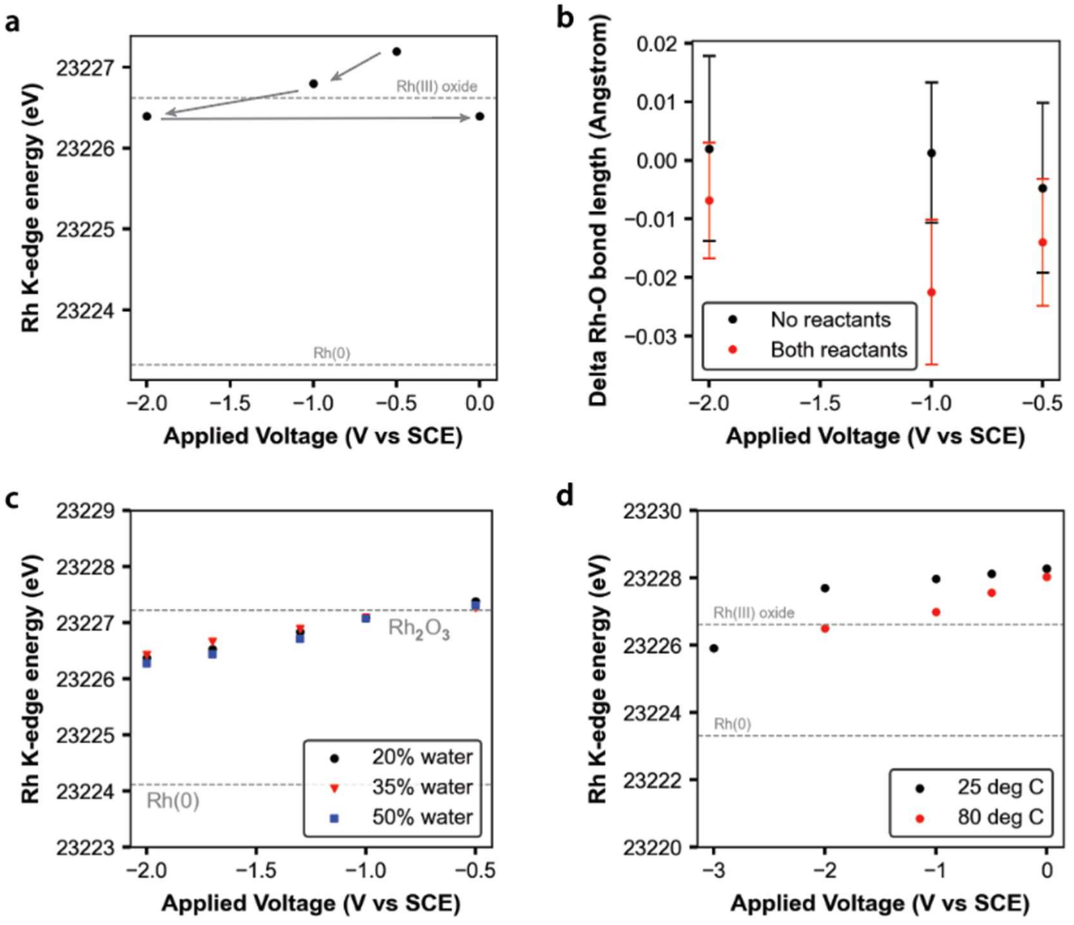


**Figure S17.** Additional XANES and EXAFS data. **a**, Plot of Rh K-edge energy as a function of applied potential, with no reactants present, at 25ºC. Grey dotted lines indicated energies of known Rh standards and grey arrows indicate temporal sequence of the tested potentials (all points shown were tested in the same cell without changing electrode or electrolyte between different potentials). **b**, fitted EXAFS data of the Rh-O coordination number both with and without reactants (i.e., CO and styrene) present at 25ºC. Error bars represent fitting errors reported by the fitting software. **c**, Effect of solvent composition on XANES response. Data collected with no styrene and no CO present at 25ºC. **d**, Effect of temperature on XANES response. Data collected with no styrene and no CO present. All data collected at ambient pressure, 0.1 M TBAOTf, 25 mM HOTf in 80% v/v IPA with 20% v/v $H_2O$ unless explicitly labeled otherwise.

## 2.8 Control tests on the robustness of electrochemical rate data

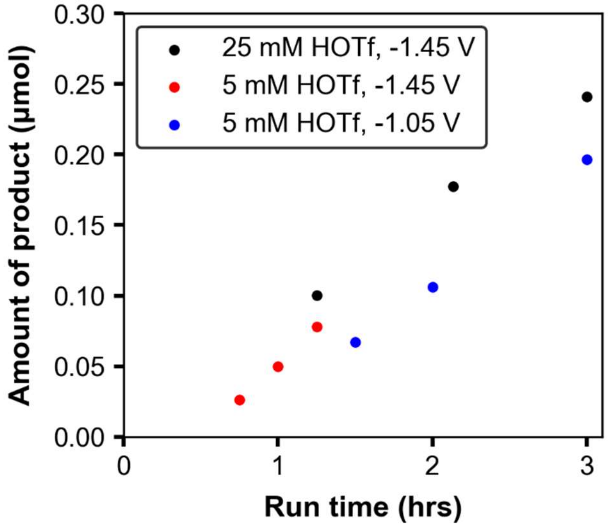


**Figure S18.** Time dependence of reaction kinetics experiments. Each point represents a different electrolysis experiment. Points represent singlicate experiments that are the replicates of corresponding averages shown in main text Figure 3. Conditions: 25ºC, 1 bar CO, 0.52 M styrene, 50% v/v IPA/$H_2O$ mixture, 0.1 M TBAOTf, and 25 mM HOTf.

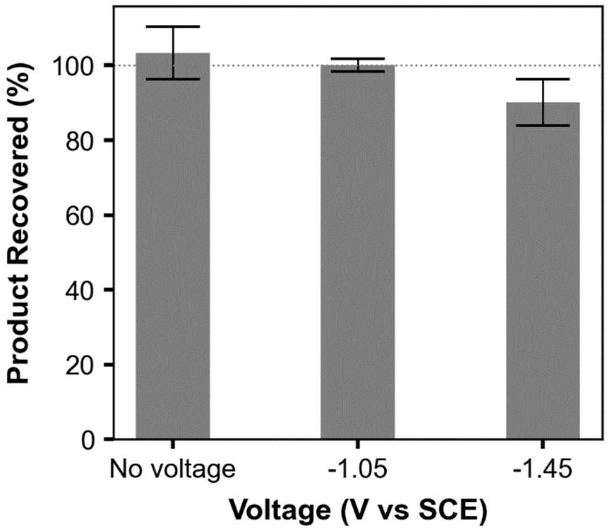


**Figure S19.** Product degradation experiments. For these experiments, a known amount of 2-phenylpropanal was added to an electrolyte solution, which was then added into an electrochemical cell. Then, under $N_2$ flow, the cell was left to sit (No voltage), or the working electrode was set to a given potential (either –1.45 or –1.05 V vs SCE). Shown is the percentage of product that was recovered after the experiment. Conditions: 25ºC, 1 bar $N_2$, 0.52 M styrene, 50% v/v IPA/$H_2O$ mixture, 0.1 M TBAOTf, and 25 mM HOTf. Error bars represent standard deviation with $n \geq 3$.

### 2.8.1 Analysis of transport limitations during kinetic measurements

The equation for transport limited current density to a planar surface is given by:

$$i_{lim} = nF\frac{D_A C_A}{\delta}$$

Where n is the number of electrons transferred in the reaction, F is Faraday's constant, $D_A$ and $C_A$ are the diffusivity and bulk concentrations of A, the most limiting reactant in the system, and δ is the hydrodynamic boundary layer thickness.

In our system, the possible limiting reactants are styrene (0.52 M), proton (0.025 M), and CO (0.009 M in pure IPA[17] and 0.0009 M in pure water at 1 atm). Taking the lowest concentration, which is CO, we will assume the solubility of pure water (which should give a lower limit). We can also assume a diffusion coefficient for CO of about 1E–5, which is a conservative estimate for gas in liquid. Finally, we can assume a boundary layer thickness of about 100 μm, which has been shown in similar cells.[11] Taken together, we get:

$$i_{lim} = 2 \cdot 96485\,\frac{(1E-5\frac{cm^2}{s})(9E-4\frac{mol}{L})(\frac{1L}{1000\ cm^3})}{1E-2\ cm} = 170\ \mu A/cm^2$$

In the kinetic data reported in main text Figure 3, the partial currents towards electro-HFN, the only CO-consuming reaction, are less than 6 μA/cm$^2$, which is less than 4% of the transport limited current density. This calculation is supported by the experimental observation that apparent reaction rate does not depend on the gas bubbling flow rate (Figure S18).

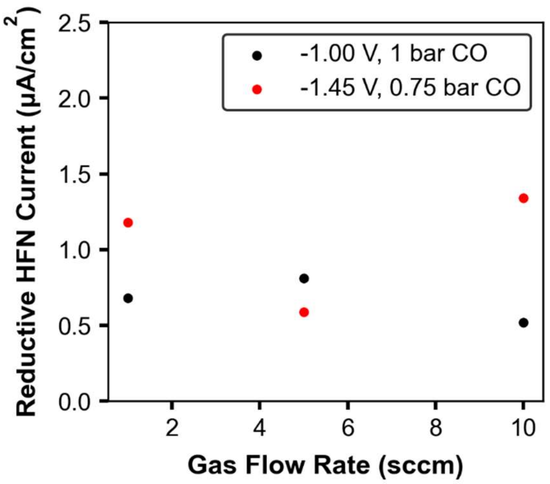


**Figure S20.** Mass transport testing via flow rate dependence. At two given conditions, partial electro-HFN current was measured as a function of gas flow rate. Since gas bubbling is a major source of convection in these electrochemical cells, an increase in rate with increased flow rate would suggest mass transport limitations. Conditions: 25°C, 1 bar $N_2$, 0.52 M styrene, 50% v/v IPA/$H_2O$ mixture, 0.1 M TBAOTf, and 25 mM HOTf.

## 2.9 Supplemental electro-HFN reaction kinetics data and observations

### 2.9.1 Styrene order dependences as a function of activity vs concentration

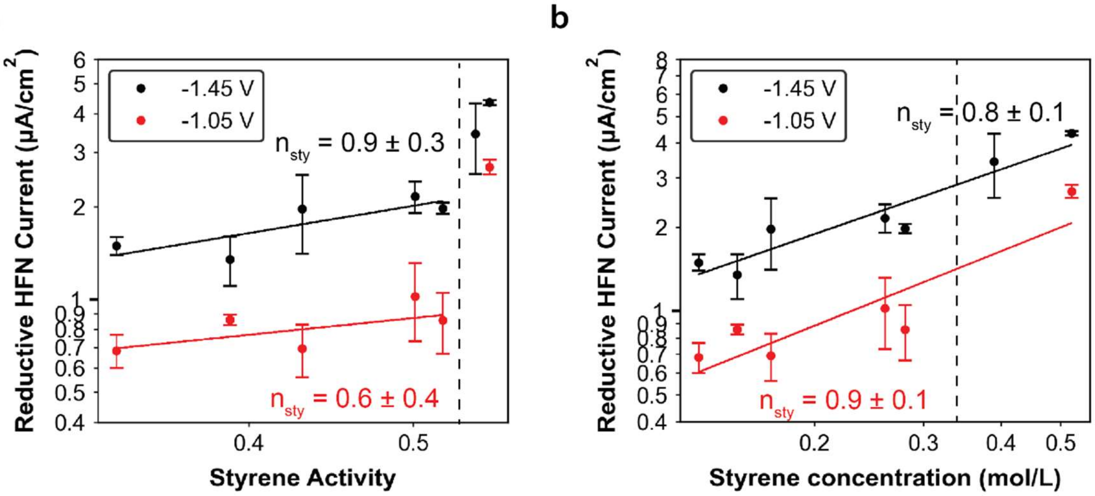


**Figure S21**. Apparent styrene order dependence as functions of **a**, activity and **b**, concentration. Solubility limit is indicated by the dashed line; experiments above the solubility limit had phase separation in the electrolyte. Conditions correspond to those in main text Figure 3.

### 2.9.2 Kinetic data within single-phase electrolytes

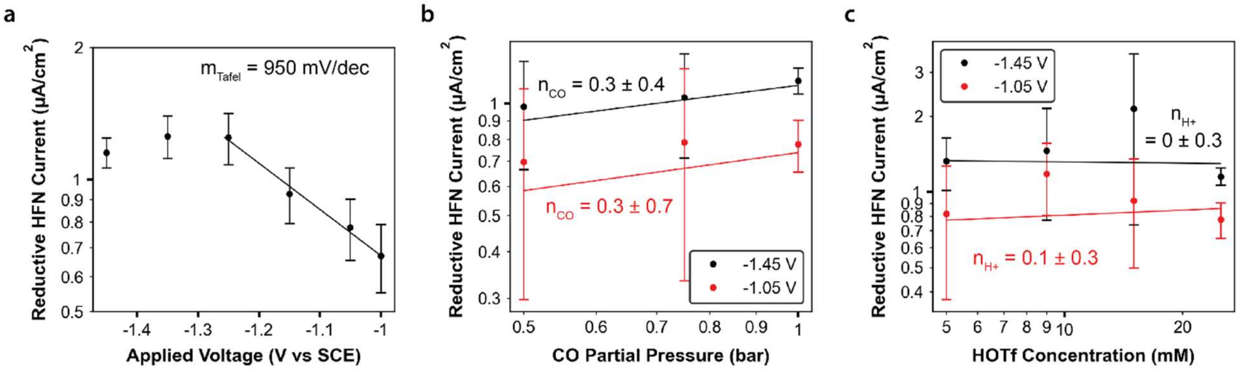


**Figure S22**. Electro-HFN order dependence data for a single-phase electrolyte system. **a**, Tafel dependence. **b**, CO dependence. **c**, proton dependence. These experiments used the same conditions as the kinetic measurements presented in main text Figure 3, with several notable exceptions. These measurements were taken using 0.28 M (rather than 0.52 M) styrene, a Pt (rather than Al) anode, and a Nafion (rather than Neosepta) membrane. Error bars represent standard deviation for $n \geq 3$.

## 2.10 Supplemental reaction kinetics data for side reactions

*Rate data for Side reactions*

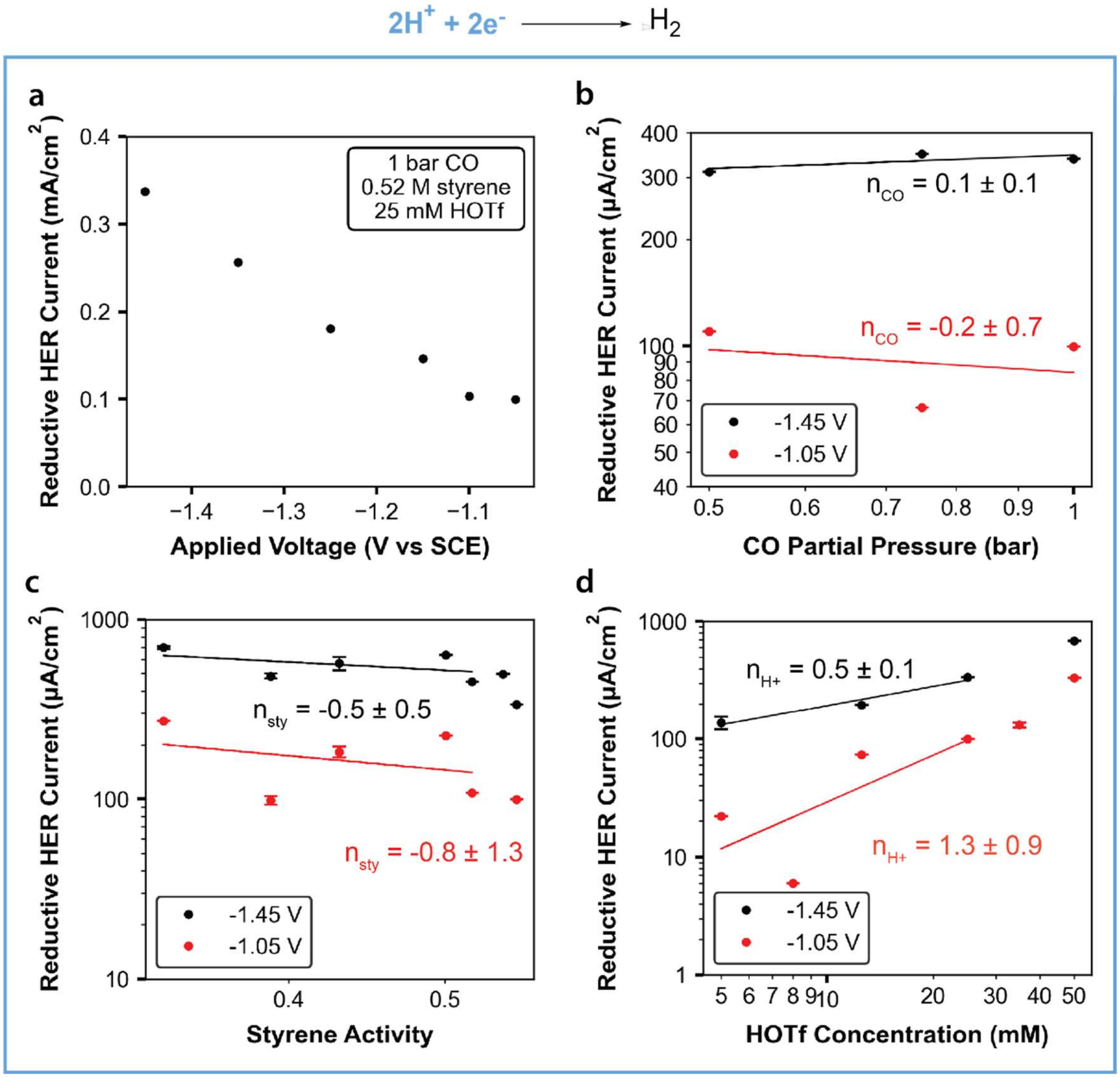


**Figure S23.** HER kinetics (corresponds to main text Figure 3). **a**, Tafel (potential) dependence. **b**, CO partial pressure dependence. **c**, styrene activity dependence. **d**, Proton (HOTf concentration) dependence. For **b-d**, black points were collected at –1.45 V vs SCE and red points at –1.05 V vs SCE. All data collected at 25ºC, 1 bar CO, 0.52 M styrene, 25 mM HOTf, 0.1 M TBAOTf in 50% v/v IPA/$H_2O$ mixture unless explicitly specified otherwise. Most points represent singlicate experiments.

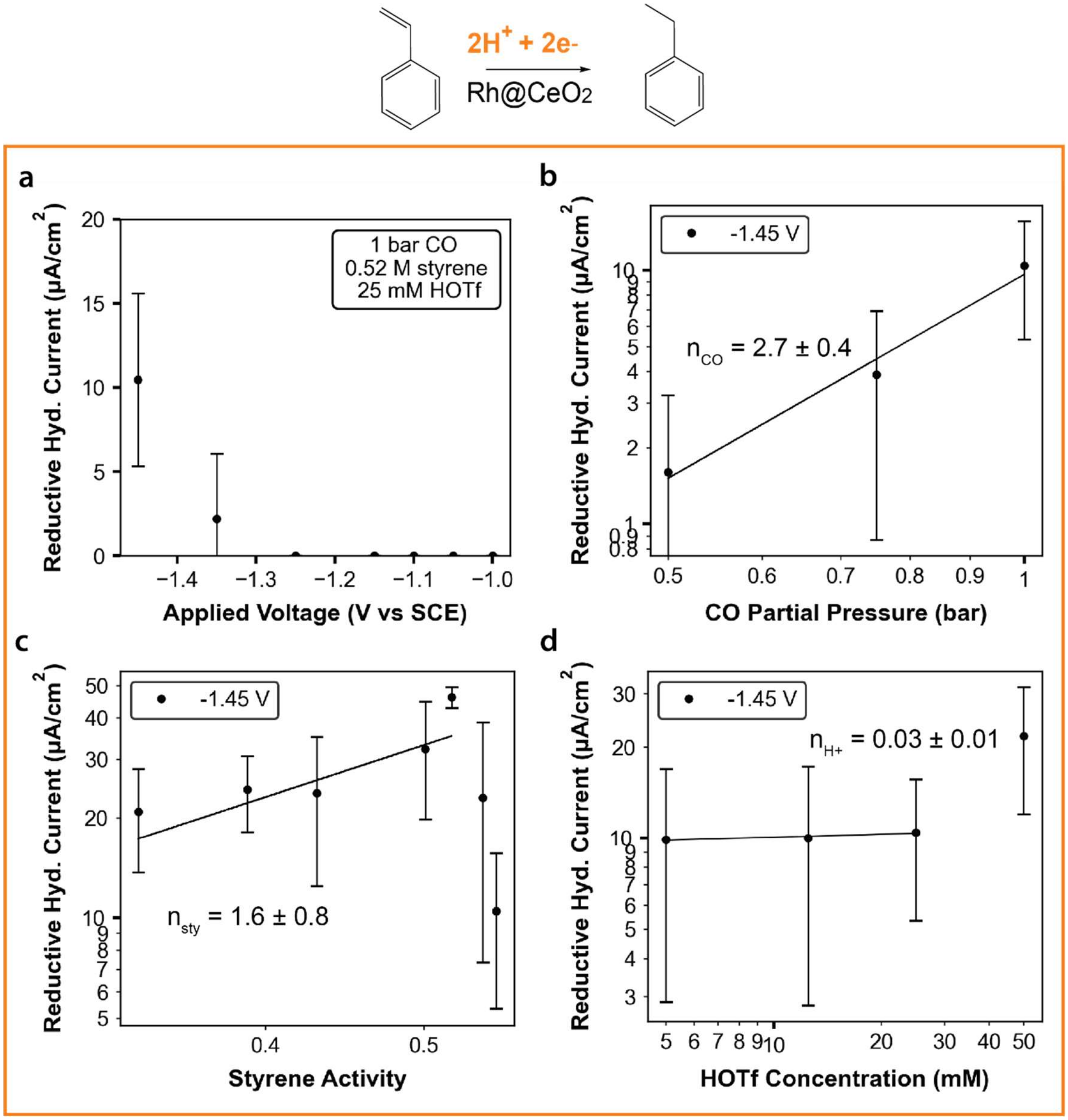


**Figure S24.** Hydrogenation kinetics (corresponds to main text Figure 3). **a**, Tafel (potential) dependence. **b**, CO partial pressure dependence. **c**, styrene activity dependence. **d**, Proton (HOTf concentration) dependence. For **b**-**d**, kinetic data is reported at –1.45 V vs SCE (no product was observed at –1.05 V vs SCE). All data collected at 25ºC, 1 bar CO, 0.52 M styrene, 25 mM HOTf, 0.1 M TBAOTf in 50% v/v IPA/$H_2O$ mixture unless explicitly specified otherwise. Error bars represent standard deviation for $n \geq 3$.

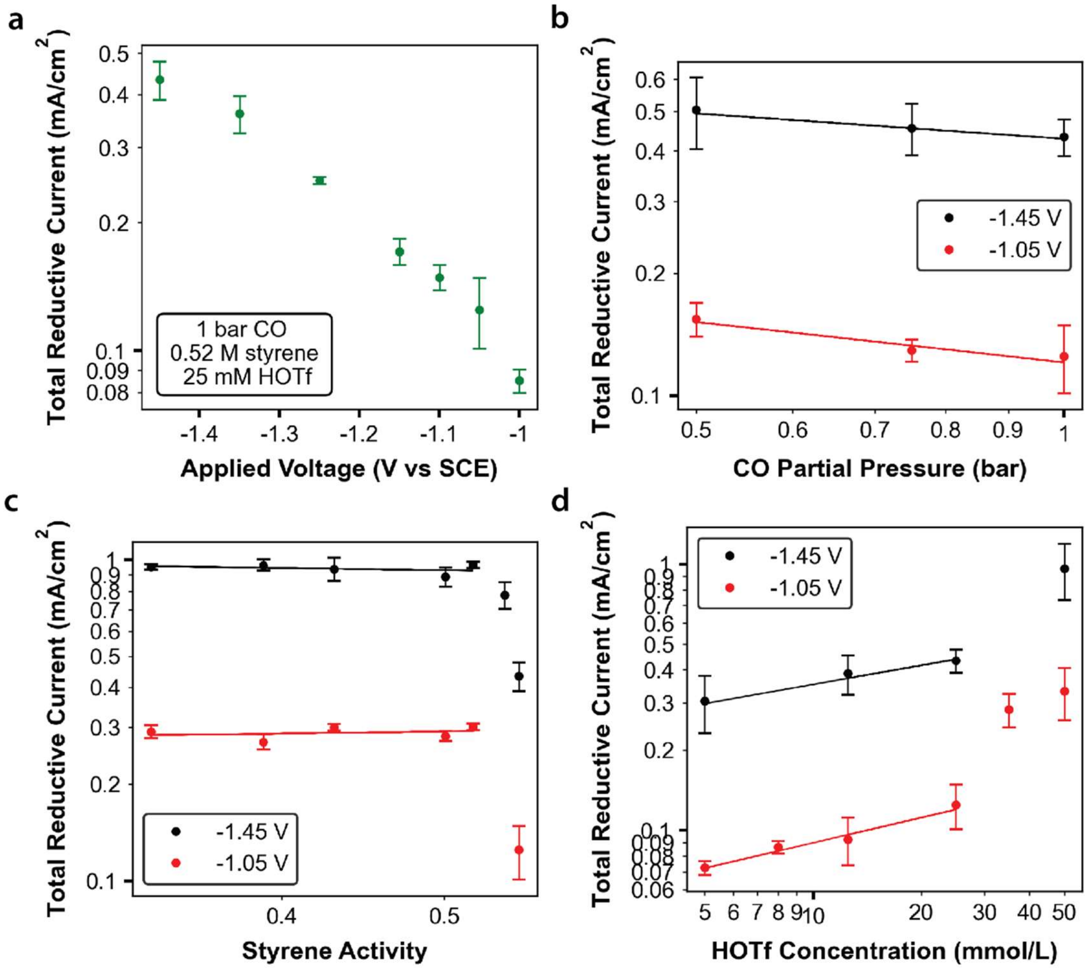


**Figure S25.** Total current kinetics (corresponds to main text Figure 3). **a**, Tafel (potential) dependence. **b**, CO partial pressure dependence. **c**, styrene activity dependence. **d**, Proton (HOTf concentration) dependence. For **b**-**d**, black points were collected at –1.45 V vs SCE and red points at –1.05 V vs SCE. All data collected at 25ºC, 1 bar CO, 0.52 M styrene, 25 mM HOTf, 0.1 M TBAOTf in 50% v/v IPA/$H_2O$ mixture unless explicitly specified otherwise.

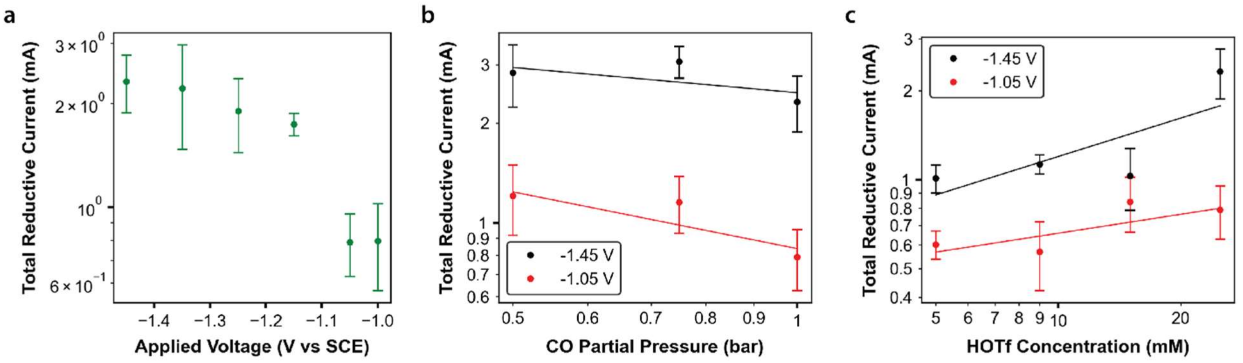


**Figure S26**. Total current kinetics for a single-phase electrolyte system (corresponds to Figure S20). **a**, Tafel dependence. **b**, CO dependence. **c**, proton dependence. These experiments used the same conditions as the kinetic measurements presented in main text Figure 3, with several notable exceptions. These measurements were taken using

0.28 M (rather than 0.52 M) styrene, a Pt (rather than Al) anode, and a Nafion (rather than Neosepta) membrane. Error bars represent standard deviation for $n \geq 3$.

## 2.11 On the conductivity of $CeO_2$ and microscopic electronic picture Rh@$CeO_2$ sites

### 2.11.1 Conductivity of $CeO_2$ and electrochemical accessibility of Rh

Our use of a $CeO_2$ support for electrocatalysis is perhaps surprising because $CeO_2$ typically has low electronic conductivity. However, $CeO_2$ is also well-known to be a highly reducible metal oxide,[18] and is also known for its ability to conduct or transport oxide ions through lattice vacancies.[19] Thus, the full description of conductivity at $CeO_2$ is actually quite complex, where depending on the composition, temperature, and degree of reducing or oxidizing conditions, ceria can be rendered electronically conducting, ionically conducting, or mixed-conducting.[20,21] We note that the references cited above study the conductivity of ceria under chemically reducing conditions at elevated temperature, so they do not directly reflect the conductivity of ceria at room temperature electrode surfaces.

Thus, another piece of evidence suggesting $CeO_2$ can be conductive enough for electrocatalysis is the fact that $CeO_2$ has previously been reported for electrochemical applications ranging from energy storage[22] to catalysis.[23] For example, for electrochemical capacitors, well-defined redox peaks associated with $Ce^{3+}/Ce^{4+}$ redox transitions have been observed for 18 – 20 nm $CeO_2$ nanocubes, and the high charge storage capacity of the materials suggests reasonable electrochemical accessibility of Ce sites along much of the electrolyte-exposed surface of the cubes.[22] In a different context, it has been shown that ostensibly non-conductive metal oxides such as $TiO_2$ can actually store electrons in electronic trap states and then deliver electron transfer equivalents to facilitate reactivity such as hydride formation and hydrogen atom transfer,[24] and that in nano-$CeO_2$, bulk reduction/oxidation processes in solution-suspended particles are also possible.[25]

Thus, it would be reasonable that at the highly reducing electrochemical conditions and for the particle size of ~20 nm reported in this work, ceria is able to facilitate electron hopping that delivers electrons fast enough for electrocatalysis.

In terms of tangible experimental evidence, from our XANES data, we do observe noticeable reduction of Rh sites under applied potential when no reactants (i.e., CO and styrene) are present (main text Figure 2b, black circles). The fact that we do see electrochemical Rh reduction (under any condition) suggests to us that some reasonable fraction of the Rh sites on our Rh@$CeO_2$ catalyst are electrochemically accessible. We do acknowledge that the timescale of the XANES measurement is on the order of minutes, whereas the relevant timescale of catalysis, according to the estimated TOFs, is on the order of seconds, so this evidence does not fully resolve the ambiguity of whether the Rh sites on Rh@$CeO_2$ are electrochemically accessible within the timescales of catalysis. Nonetheless, it does preclude a molecular picture in which ceria is completely non-conductive and obstructs electron transfers to most of the Rh sites.

We have also attempted to titrate electrochemically accessible Rh sites via CO stripping experiments. Unfortunately, we were not able to observe well-defined redox peaks associated with electrochemical CO stripping. However, this is likely due to the fact that binding interactions between $Rh^{3+}$ and CO are not as well-defined as those between metallic Rh (where most/all of the CO stripping literature precedent lies)[26,27] and CO.[28]

Finally, we replaced the Rh@$CeO_2$ catalyst with more conductive $Rh_2O_3$ nanoparticles. The electronic classification of $Rh_2O_3$ ranges from semimetal to semiconductor.[29,30] We did not see any apparent improvements in electro-HFN partial current for $Rh_2O_3$ electrodes with the same total Rh mass content (Figure S27b), though we do note that in the $Rh_2O_3$ nanoparticle case, not all Rh atoms would be surface

accessible. Thus, it is likely that for both Rh@$CeO_2$ and $Rh_2O_3$ catalyst formulations, there remains opportunity to increase the percentage of deposited Rh sites participating in catalysis.

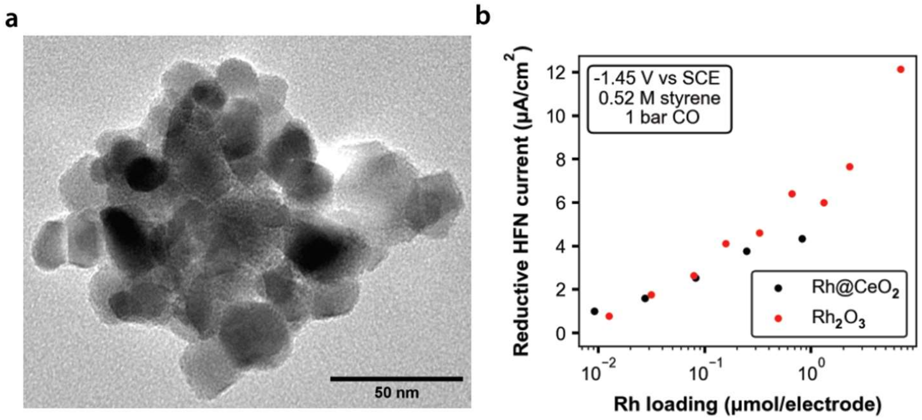


**Figure S27**. Details for $Rh_2O_3$ catalyst extension. **a**, TEM image of commercially available $Rh_2O_3$ nanoparticles. **b**, Electro-HFN reactivity for electrodes with either a Rh@$CeO_2$ or $Rh_2O_3$ catalyst, at different total Rh contents. Typical potentiostatic electrolysis conditions were used: –1.45 V vs SCE, 1 bar CO, 25ºC, 0.52 M styrene, 25 mM HOTf, 0.1 M TBAOTf in 50/50 v/v IPA/$H_2O$.

### 2.11.2 Possible effects of reactants on *operando* XANES response

This discussion will touch on possible phenomena that could explain the trends seen in main text Figure 2b. As a short summary of the observation, the response of Rh valency on applied potential depends on which reactants are present, where when neither CO nor styrene is present, we observe a strong potential-dependent response, but CO attenuates the response, and styrene almost completely eliminates it.

First, it could be that CO/styrene chemisorption shifts the Rh redox potential to become more negative, so Rh reduction is simply less thermodynamically favorable upon reactant coordination. Alternatively, perhaps CO or styrene coordination changes the kinetics of Rh reduction, where perhaps some strongly coordinated ligand in the Rh inner sphere increases the reorganization energy associated with electron transfer (ET), and thus causes Rh reduction to become more kinetically challenging. Finally, it could be that upon interacting with styrene or CO, the most accessible orbital for receiving an ET is no longer Rh-centered, but instead ligand-centered. Thus, even if an ET transfer event did occur, the electron would be rapidly transferred to styrene or CO, leaving the Rh oxidation state unchanged. Stated slightly differently, these reactants could act as "sinks" for electron (and proton) equivalents that would otherwise go towards reducing Rh. At this point we are unable to exclude any hypotheses to favor only one of the above explanations."

## 2.12 On possible reaction mechanisms

In Figure S28a, we present a general and speculative interpretation of the kinetic data. In short, there appears to be a potential-dependent shift in the most abundant reactive intermediate (MARI) from some species $I_0$ to another species $I_1$. The CO and Tafel dependencies indicate that CO and electron should participate prior to the formation of $I_1$ (voltage-dependent saturation behavior at high overpotential ($\eta$)) . The role of styrene and protons are more ambiguous, as discussed in the main text. If the styrene and proton dependencies are interpreted without considering second-order effects, they are consistent with a mechanism where styrene participates after formation of $I_1$ (first order dependence irrespective of $\eta$), and where protons do not participate in any kinetically relevant steps (weak-to-no dependence irrespective of $\eta$).

Specific catalytic cycles that can be mapped to Figure S28a are shown in Figures S28b and S28c. These are not exhaustive nor intended to be mechanistic proposals, but rather to clarify potential species that could correspond to the general scheme presented in Figure S28a. Notably, both of these mechanisms are inconsistent with the apparent proton order dependence data: below we suggest that the participation of electrons in the catalytic cycle would be towards the generation of a Rh-hydride species, but we do not observe strong or any dependence of reaction rate on proton concentration. Beyond the inherent variability in the proton dependence data, this inconsistency may also be due to some complex role of protons that might affect, for example, the double layer structure. We do not at this time have a full mechanistic picture that accounts for all of our experimental observations.

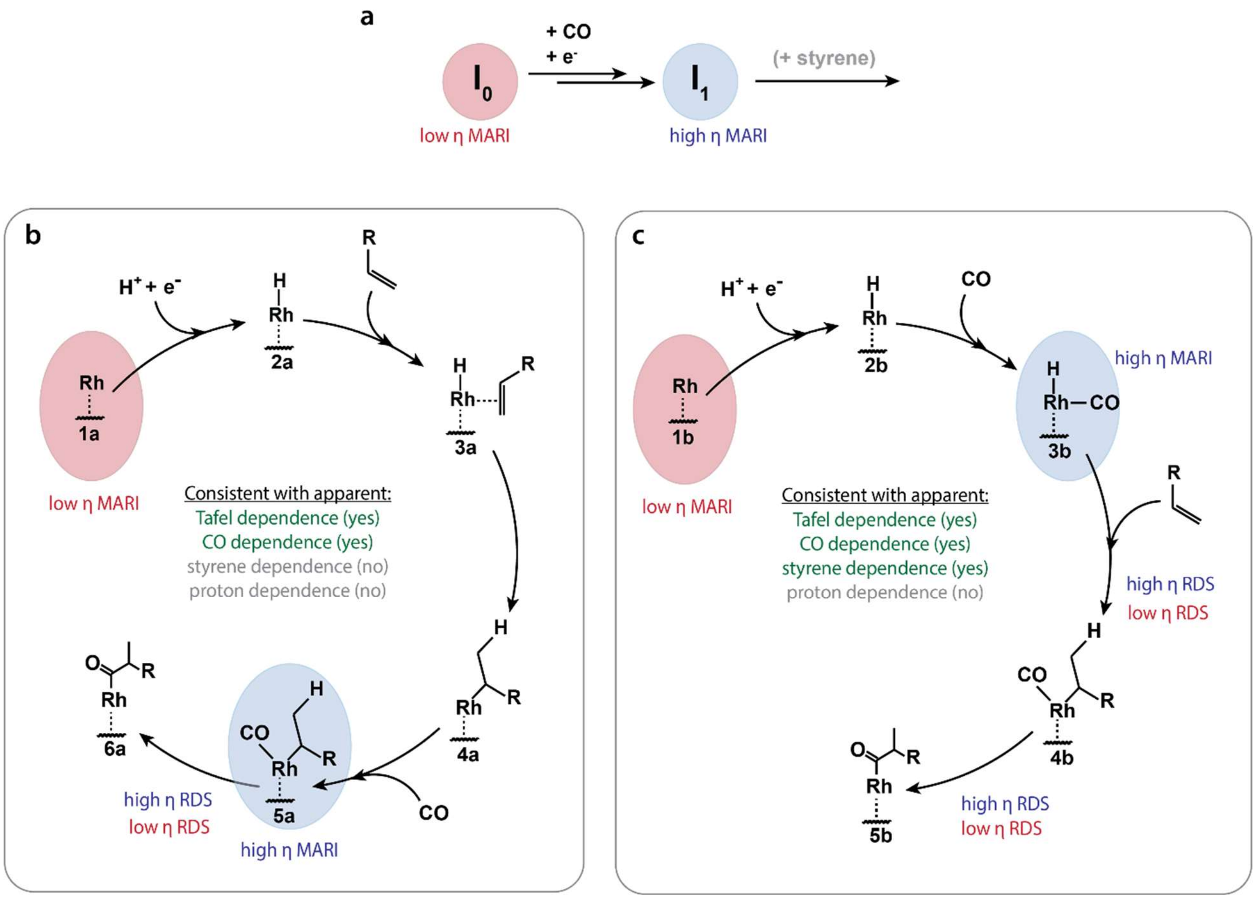


**Figure S28**. Potential mechanisms corresponding to the kinetic data. **a**, an overall schematic of the general type of mechanism that is consistent with potential-dependent CO and Tafel saturation. Since the role of styrene is less clear

from the kinetic data, a possible interpretation consistent with the rate data is indicated in grey and within parentheses. **b**, possible mechanism, based closely on the chemistry of the thermo-HFN mechanism, that is consistent with the CO and Tafel data, but cannot explain the observed styrene and proton dependencies. **c**, possible mechanism that is consistent with the apparent CO, Tafel, and styrene dependencies, but cannot explain the observed proton dependence. Species invoked as the most abundant reactive intermediate (MARI) are highlighted in blue/red, and steps that could be the corresponding rate determining step (RDS) are labeled with red/blue.

*Alternative interpretation of Tafel dependence*

We also note that if the electronic structure of $CeO_2$ is formulated as a semiconductor, it can be the case that depletion of charge carriers within the $CeO_2$ support could lead to apparent saturation behavior in the Tafel dependence (e.g., movement of electronic charges within the solid electrode becomes "transport limited").[31] Thus, the curvature that we see in the Tafel slope, which we ascribe to the buildup of a surface intermediate, may also be explained by this phenomenon.

## 2.13 Overpotentials and energy efficiency of electro-HFN

We will first require a calculation of the expected equilibrium potential for the electro-HFN half reaction. For this, let us consider a representative, gas-phase thermochemical hydroformylation reaction, for which all of the gas-phase thermochemistry data is readily available:[32]

Ethylene + CO + $H_2$ → Propionaldehyde $\qquad \Delta G^{\text{o}}_{\text{tHFN}}$

Where the standard Gibbs free energy of reaction is given by $\Delta G^{\text{o}}_{\text{tHFN}}$. This reaction can be written as two electrochemical half-reactions, the electro-HFN half reaction and the hydrogen oxidation (HOR) half reaction:

Ethylene + CO + $2H^+$ + $2e^-$ → Propionaldehyde $\qquad E^{\text{o}}_{\text{eHFN}}$

$H_2$ → $2H^+$ + $2e^-$ $\qquad E^{\text{o}}_{\text{HOR}} \stackrel{\text{def}}{=} 0\,V\ vs\ SHE$

Where each half reaction is associated with a standard equilibrium potential. For HOR, the standard equilibrium potential is, by definition, the standard hydrogen electrode (SHE). The relationship between all of these quantities is given by the equation:

$$\Delta G = -nFE_{cell}$$

Where in this case, we have:

$$\Delta G^{\text{o}}_{\text{tHFN}} = -nF * (E^{\text{o}}_{\text{HOR}} - E^{\text{o}}_{\text{eHFN}})$$

$$\Delta G^{\text{o}}_{\text{tHFN}} = -\left(2\,\frac{mol\ e}{mol\ rxn}\right) * \left(96485\,\frac{C}{mol\ e}\right) * \left(-E^{\text{o}}_{\text{eHFN,vs SHE}}\right)$$

Calculating the Gibbs free energy of reaction from gas-phase thermochemical data gives:

$$\Delta G^{\text{o}}_{\text{tHFN}} = -58.44\ kJ/mol$$

And solving for the standard equilibrium potential of electro-HFN gives:

$$E^{o}_{\text{eHFN,vs SHE}} = +\,0.303\,V\;vs\;SHE$$

Since electro-HFN has an equal number of protons and electrons, the pH dependence of the actual equilibrium potential under different pH conditions is given by:

$$E_{\text{eHFN}} = E^{o}_{\text{eHFN}} - 0.059 * pH = (+\,0.303\,V - 0.059 * \text{pH})\ \text{V vs SHE}$$

This value represents the equilibrium potential for electro-HFN (even within the liquid phase electrolyte, due to gas-liquid equilibrium) of ethylene under 1 atm ethylene and 1 atm of CO. We will use this as an approximation for the thermodynamics of styrene electro-HFN as well.

Under typical operating, conditions, we add 25 mM of HOTf as explicit acid. To get a rough estimate of equilibrium potential, we will make a coarse assumption that proton activity within the electrolyte reflects acid concentration. Thus, we get:

$$E_{\text{eHFN}} = \left(+0.303V - 0.059 * -\log_{10}(0.025)\right) = +0.21\ \text{V vs SHE} = -0.04\ \text{V vs SCE}$$

With respect to this analysis, we provide several points of discussion:

First, the positive standard equilibrium potential of electro-HFN with respect to SHE means that electro-HFN can be thermodynamically feasible in conditions where hydrogen evolution (HER) is not. Thus, direct electro-HFN reactions could, in principle, save energy by bypassing $H_2$ as an intermediate. Specifically, a direct electro-HFN reaction using water as the H atom source would thermodynamically require less cell potential than an alternative process that uses water splitting to generate $H_2$ and then feeds the $H_2$ gas to a thermo-HFN reaction.

Second, for experiments performed potentiostatically at 1 bar CO, our typical operating conditions in this work are around –1.05 to –1.45 V vs SCE, which reflect about 1 to 1.5 V of overpotential with respect to the thermodynamics of electro-HFN, and 0.7 to 1.2 V of overpotential with respect to the thermodynamics of hydrogen evolution. Thus, further optimization of this chemistry will be required to lower these overpotentials, and will be the subject of future work.

# 3. References


1. Amsler, J. *et al.* Prospects of Heterogeneous Hydroformylation with Supported Single Atom Catalysts. *J Am Chem Soc* **142**, 5087–5096 (2020).

2. Vengatesan, S., Cho, E., Kim, H.-J. & Lim, T.-H. Effects of curing condition of solution cast Nafion® membranes on PEMFC performance. *Korean J. Chem. Eng* **26**, 679–684 (2009).

3. Ro, I., Xu, M., Graham, G. W., Pan, X. & Christopher, P. Synthesis of Heteroatom Rh-ReOx Atomically Dispersed Species on Al2O3 and Their Tunable Catalytic Reactivity in Ethylene Hydroformylation. *ACS Catal* **9**, 10899–10912 (2019).

4. Leshchev, D. *et al.* The Inner Shell Spectroscopy beamline at NSLS-II: a facility for in situ and operando X-ray absorption spectroscopy for materials research. *J Synchrotron Radiat* **29**, 1095–1106 (2022).

5. Ravel, B. & Newville, M. ATHENA, ARTEMIS, HEPHAESTUS: data analysis for X-ray absorption spectroscopy using IFEFFIT. *J Synchrotron Radiat* **12**, 537–541 (2005).

6. Williams, K., Limaye, A., Weiss, T., Chung, M. & Manthiram, K. Accounting for species' thermodynamic activities changes mechanistic interpretations of electrochemical kinetic data. *ChemRxiv* (2022) doi:10.26434/CHEMRXIV-2022-VK5Z9.

7. Corbin, N., Zeng, J., Williams, K. & Manthiram, K. Heterogeneous molecular catalysts for electrocatalytic CO 2 reduction. *Nano Res* **12**, 2093–2125 (2019).

8. Chu, A. T. & Surendranath, Y. Aprotic Solvent Exposes an Altered Mechanism for Copper-Catalyzed Ethylene Electrosynthesis. *J Am Chem Soc* **144**, 5359–5365 (2022).

9. Roberts, J. A. S. & Bullock, R. M. Direct determination of equilibrium potentials for hydrogen oxidation/production by open circuit potential measurements in acetonitrile. *Inorg Chem* **52**, 3823–3835 (2013).

10. Lazzaroni, R., Raffaelli, A., Settambolo, R., Bertozzi, S. & Vitulli, G. Regioselectivity in the rhodium-catalyzed hydroformylation of styrene as a function of reaction temperature and gas pressure. *Journal of Molecular Catalysis* **50**, 1–9 (1989).

11. Williams, K. *et al.* Protecting effect of mass transport during electrochemical reduction of oxygenated carbon dioxide feedstocks. *Sustain Energy Fuels* **3**, 1225–1232 (2019).

12. Kutsche, I., Gildehaus, G., Schuller, D. & Schumpe, A. Oxygen solubilities in aqueous alcohol solutions. *J Chem Eng Data* **29**, 286–287 (1984).

13. Hori, Y. Electrochemical CO 2 Reduction on Metal Electrodes. *Modern Aspects of Electrochemistry* 89–189 (2008) doi:10.1007/978-0-387-49489-0_3.

14. Zhang, J. *et al.* Efficient CO Electroreduction to Methanol by CuRh Alloys with Isolated Rh Sites. *ACS Catal* **13**, 7170–7177 (2023).

15. Solubilities of Gases in Water at 293 K. https://www.wiredchemist.com/chemistry/data/solubilities-gases.

16. Wainwrlght, M. S., Ahn, T., Trimm, D. L. & Cant, N. W. Solubility of Hydrogen in Alcohols and Esters. *J. Chem. Eng. Data* **32**, 22–24 (1987).

17. Tenner, S. P., Walnwright, M. S., Trlmm, D. L. & Cant, N. W. Solubility of Carbon Monoxide in Alcohols. *J Chem Eng Data* **28**, 59–61 (1983).

18. Pinto, F. M., Suzuki, V. Y., Silva, R. C. & La Porta, F. A. Oxygen Defects and Surface Chemistry of Reducible Oxides. *Front Mater* **6**, 260 (2019).

19. Andersson, D. A., Simak, S. I., Skorodumova, N. V., Abrikosov, I. A. & Johansson, B. Optimization of ionic conductivity in doped ceria. *Proceedings of the National Academy of Sciences* **103**, 3518–3521 (2006).

20. Tuller, H. L. & Nowick, A. S. Defect Structure and Electrical Properties of Nonstoichiometric CeO2 Single Crystals. *J Electrochem Soc* **126**, 209–217 (1979).

21. Chiang, Y. M., Lavik, E. B. & Blom, D. A. Defect thermodynamics and electrical properties of nanocrystalline oxides: pure and doped CeO2. *Nanostructured Materials* **9**, 633–642 (1997).

22. Chavhan, M. P., Som, S. & Lu, C.-H. Size-controlled ceria nanocubes obtained via hydrothermal route for electrochemical capacitors. *Mater Lett* **257**, 126598 (2019).

23. Song, X. Z., Zhu, W. Y., Wang, X. F. & Tan, Z. Recent Advances of CeO2-Based Electrocatalysts for Oxygen and Hydrogen Evolution as well as Nitrogen Reduction. *ChemElectroChem* **8**, 996–1020 (2021).

24. Peper, J. L., Gentry, N. E., Boudy, B. & Mayer, J. M. Aqueous TiO2Nanoparticles React by Proton-Coupled Electron Transfer. *Inorg Chem* **61**, 767–777 (2022).

25. Damatov, D. *et al.* Redox Reactivity of Colloidal Nanoceria and Use of Optical Spectra as an in Situ Monitor of Ce Oxidation States. *Inorg Chem* **57**, 14401–14408 (2018).

26. Moniri, S., Van Cleve, T. & Linic, S. Pitfalls and best practices in measurements of the electrochemical surface area of platinum-based nanostructured electro-catalysts. *J Catal* **345**, 1–10 (2017).

27. Montero, M. A., Gennero De Chialvo, M. R. & Chialvo, A. C. Kinetics of the hydrogen oxidation reaction on nanostructured rhodium electrodes in alkaline solution. *J Power Sources* **283**, 181–186 (2015).

28. Bae, G. *et al.* Quantification of Active Site Density and Turnover Frequency: From Single-Atom Metal to Nanoparticle Electrocatalysts. *JACS Au* **1**, 586–597 (2021).

29. Leiva, H., Kershaw, R., Dwight, K. & Wold, A. Magnetic and electrical properties of rhodium(III)oxide(III). *Mater Res Bull* **17**, 1539–1544 (1982).

30. Roy, A. & Ghose, J. Electrical and Magnetic Characterization of Rh2O3-I. *Mater Res Bull* **33**, 547–551 (1998).

31. Bockris, J., Reddy, A. & Gamboa-Aldeco, M. *MODERN ELECTROCHEMISTRY SECOND EDITION Fundamentals of Electrodics*. vol. Volume 2A (2002).

32. NIST WebBook. https://webbook.nist.gov/.
